# Supplementary material for: Picornavirus VP2 protein suppresses innate immunity through selective autophagic degradation of IKBKE/IKKε
Source: Autophagy. 2025 Dec 14;22(2):330–50. doi: 10.1080/15548627.2025.2597460 (PMC12834145; doi:10.1080/15548627.2025.2597460)
Supplement: Manuscirpt track change.pdf [file KAUP_A_2597460_SM2515.pdf]

Picornavirus VP2 protein suppresses innate immunity through selective autophagic degradation of IKBKE/IKKε

Kangli Li[a,b,#], Xiangle Zhang[a,b,#], Dandan Dong[a,b], Boning Zhu[a,b], Shuo Wang[d], Xiaodan Wen[a,b], Weijun Cao[a,b], Yi Ru[a,b], Hong Tian[a,b], Guoliang Zhu[a], Jijun He[a,b], Jianhong Guo[a,b], Jianye Dai[d], Haixue Zheng[a,c,]*, Fan Yang[a,]*, Zixiang Zhu[a,]*

[a] State Key Laboratory for Animal Disease Control and Prevention, College of Veterinary Medicine, Lanzhou University, Lanzhou Veterinary Research Institute, Chinese Academy of Agricultural Sciences, Lanzhou 730046, China

[b] WOAH/National reference laboratory for foot-and-mouth disease, Lanzhou 730046, China

[c] Gansu Province Research Center for Basic Disciplines of Pathogen Biology, Lanzhou 730046, China.

[d] School of Pharmacy, Lanzhou University, 199 West Donggang Rd, Lanzhou 730000, China.

# These authors contribute equally to this study.

*Corresponding authors.

Email addresses: zhuzixiang@caas.cn (Z. Zhu); yangfan02@caas.cn (F. Yang); haixuezheng@163.com (H. Zheng)

ABSTRACT

Senecavirus A (SVA) belongs to the picornaviruses and has emerged as a promising candidate for oncolytic virotherapy in humans. Understanding the immune suppression mechanisms employed by SVA can help optimize its therapeutic efficacy as an oncolytic virus while simultaneously minimizing its immune suppressive effects on normal tissues. In this study, we identified a novel function of the SVA structural protein VP2 as a key viral immune suppressive factor during SVA infection. VP2 targets and degrades IKBKE/IKKε, a key component of the innate immune pathway, thereby suppressing host innate immune responses. It preferentially interacts with the selective autophagic receptor CALCOCO2/NDP52 (calcium binding and coiled-coil domain 2), which then recognizes the K33-linked ubiquitinated IKBKE and delivers it to phagophores for degradation. The E3 ligase RNF114 is responsible for catalyzing the K33-linked ubiquitination of IKBKE at Lys490, and VP2 significantly promoted this modification, which further accelerated IKBKE degradation. Importantly, we found that picornavirus VP2 proteins share this conserved mechanism in degradation of IKBKE and suppression of host innate immunity. These data elucidate the negative regulatory mechanism involving the VP2-RNF114-IKBKE/IKKε-CALCOCO2 axis, and reveal an immune evasion strategy employed by picornaviruses. These findings will provide valuable insights for the development of picornaviral vaccines and antiviral/antitumor therapeutics.

Abbreviations: 3-MA: 3-methyladenine; ATG5: autophagy related 5; ATG7: autophagy related 7; CALCOCO2/NDP52: calcium binding and coiled-coil domain 2;

CQ: chloroquine; Coco-IP: co-immunoprecipitation; DAPI: 4 ' ,6-diamidino-2 ' -phenylindole; EV71: enterovirus 71; FMDV: foot-and-mouth disease virus; hpi: hours post-infection; IFN: interferon; IKBKE/IKKε: inhibitor of nuclear factor kappa B kinase subunit epsilon); ISGs: IFN-stimulated genes; MAP1LC3/LC3: microtubule associated protein 1 light chain 3; MG132: cbz-leu-leu-leucinal; MOI: multiplicity of infection; NBR1: NBR1 autophagy cargo receptor; OPTN: optineurin; RNF114: ring finger protein 114; RT-PCR: real-time polymerase chain reaction; siRNA: small interfering RNA; SQSTM1/p62: sequestosome 1; SVA: Senecavirus A; $TCID_{50}$: 50% tissue culture infectious doses. TOLLIP: toll interacting protein; TRIM17: tripartite motif containing 17; TRIM25: tripartite motif containing 25; TRIM28: tripartite motif containing 28; TRIP12/THRI12: thyroid hormone receptor interactor 12; Ub: ubiquitin; Vec: vector; WCL: whole-cell lysate; WT: wild-type.

KEYWORDS Autophagy; CALCOCO2/NDP52; IKBKE/IKKε; innate immune response; NDP52; picornavirus; RNF114; viral replication; VP2 protein

Introduction

Senecavirus A (SVA) is an emerging virus that belongs to the genus *Senecavirus* within the family *Picornaviridae* [1, 2]. Great importance has been attached to SVA due to its oncolytic potential, as it selectively infects human tumor cells and exhibits promising therapeutic effects against various cancers, positioning it as a candidate for human oncolytic virotherapy [3, 4]. Additionally, SVA infects pigs, causing vesicular disease with symptoms resembling those of foot-and-mouth disease virus (FMDV)

and other vesicular viruses. This similarity complicates the diagnosis and control of vesicular diseases in swine populations. SVA genome is composed of a single-stranded positive-sense RNA, approximately 7.2 kb in length. It contains a 5′ untranslated region (5′ UTR), a large open reading frame (ORF), and a 3′ UTR [5, 6]. The ORF encodes four structural proteins (VP4, VP2, VP3, and VP1) and seven non-structural proteins (2A, 2B, 2C, 3A, 3B, 3C, and 3D), playing multiple sophisticated regulatory functions during viral replication and infection [7].

Innate immunity serves as the first line of defense against viral infections. Upon RNA virus infections, the cellular pathogen recognition receptors RLRs (RIGI and/or IFIH1/MDA5) sense viral genomes. The CARD domains of RLRs are then exposed and interact with the CARD domains of MAVS/VISA/IPS-1. Following this interaction, TANK, TRAF3 and TRAF6 are recruited, which subsequently activates distinct signaling pathways by engaging CHUK/IKKα, IKBKEB/IKKβ or TBK1 and IKK-εIKBKE. CHUK/IKBKB IKK-α/β then activates NFKBIA/IκBα, leading to the release of the NFKB1/p50-RELA/p65 complex and the subsequent expression of various proinflammatory cytokines. Meanwhile, TBK1 and IKK-εIKBKE phosphorylate IRF3 or IRF7, promoting the formation of IRF3-IRF3 or IRF3-IRF7 dimers, which in turn initiate the production of type I interferons (IFNs) [8]. However, to ensure their survival and replication within the host cells, many viruses have evolved multiple strategies to disrupt this innate immune barrier and suppress host innate immune response.

In eukaryotic cells, macroautophagy (commonly referred to as autophagy) is a

highly conserved homeostatic process that allows cells to sequester damaged organelles, dysfunctional proteins, and invading pathogens, and subsequently deliver them to lysosomes for degradation [9]. This process is highly selective, with various cargo receptors targeting specific substrates and recognizing distinct degradation signals. These signals are primarily ubiquitinated substrates, which are selectively transported to autophagosomes for degradation [10, 11]. The cargo receptors such as SQSTM1/p62 (sequestosome 1), NBR1 (NBR1 autophagy cargo receptor), OPTN (optineurin), CALCOCO2/NDP52 (calcium binding and coiled-coil domain 2), and TOLLIP (toll interacting protein) play a crucial role in this process. They possess both ubiquitin-binding domains/UBDs and LC3-interacting regions/LIRs, which enable them to recognize and deliver ubiquitinated substrates to phagophores for subsequent degradation [12, 13]. Many viruses have evolved sophisticated strategies to exploit selective autophagy to degrade innate immune adaptor molecules, thereby suppressing innate immunity and promoting their own replication [14]. For instance, the NSP13 protein of SARS-CoV-2 recruits TBK1 for autophagic degradation via the selective autophagy receptor SQSTM1, thereby inhibiting the production of type I IFN and facilitating viral replication [15]. Similarly, the VP3 protein of infectious bursal disease virus (IBDV) induces TRAF6 autophagic degradation in an SQSTM1-dependent manner, inhibiting the activation of the IFN signaling pathway and enhancing viral replication [16]. The PB1 protein of influenza A virus (IAV) manipulates NBR1-mediated selective autophagy to degrade MAVS, thereby suppressing the innate immune response [17]. Additionally, the UL21 protein of

alpha-herpesvirus degrades CGAS through TOLLIP-mediated selective autophagy, thus inhibiting innate immunity [18].

Previous studies have demonstrated that various picornaviruses enhance viral replication by inducing cellular autophagy and blocking innate immunity. Such as, FMDV VP1 degrades YTHDF2 through the autophagy pathway, thereby regulating IRF3 activity and promoting viral replication [19]. FMDV VP3 induces autophagy through the TP53-BAD-BAX axis, which facilitates viral replication [20]. In addition, FMDV VP3 interacts with HDAC8, promoting its autophagic degradation and thereby facilitating viral replication [21]. FMDV VP2 interacts with HSPB1, activates the cellular EIF2S1-ATF4 pathway, induces autophagy, and enhances FMDV replication [22]. EV71 3D protein interacts with BECN1 (beclin 1), inducing autophagy and promoting viral replication [23]. EV71 VP1 promotes autophagy and enhances viral replication by regulating the MTOR pathway [24]. These findings highlight the diverse mechanisms by which picornaviruses manipulate autophagy to support their replication and evade host immune responses.

The intricate interplay between autophagy and immune regulation during SVA infection has been reported previously. Specifically, the SVA 2AB protein orchestrates the formation of a large protein complex with MARCHF8 and MAVS, leading to the subsequent degradation of these key proteins and thereby effectively inhibiting the type I IFN response [25]. The SVA 3C protease cleaves OPTN, thereby impeding selective autophagy and IFN production [26]. Furthermore, SVA 3C protease cleaves EPHA2, blocking MTOR activation, which in turn regulates cell death and enhances

viral replication [27]. Collectively, the previous findings demonstrate that SVA employs multiple nonstructural viral proteins and sophisticated strategies to suppress host innate immunity and facilitate its own replication. Viral structural proteins play crucial roles in various processes of viral infection, such as virus entry, protection of the viral genome, assembly and release of viral particles, and immune evasion. Moreover, they directly affect the immunogenic efficacy of vaccines. Therefore, elucidating the mechanisms by which viral structural proteins exert their functions during viral infection is of great significance for the development of antiviral drugs, oncolytic therapy strategies, and vaccines. However, the precise functions of SVA structural proteins in modulating host innate immunity and regulating autophagy remain to be fully elucidated.

In this study, we elucidated a novel mechanism by which the VP2 protein of SVA antagonizes the host innate immune response. Specifically, we identified that SVA VP2 interacts with IKBKE~~IKKε~~ and CALCOCO2 ~~NDP52~~, leading to the degradation of IKBKE~~IKKε~~ and subsequent inhibition of the type I IFN signaling pathway. Mechanistically, VP2 enhances K33-linked polyubiquitination of IKBKE~~IKKε~~ at residue K490, a process mediated by the E3 ubiquitin ligase RNF114. The ubiquitinated IKBKE~~IKKε~~ is then recognized by the selective autophagy receptor CALCOCO2~~NDP52~~ and delivered to autophagosomes for degradation. Importantly, we observed that VP2 proteins from other picornaviruses, such as foot-and-mouth disease virus (FMDV) and enterovirus 71 (EV71), similarly inhibit type I IFN signaling and promote viral replication. Picornavirus VP2 proteins share this

conserved mechanism in degrading IKBKE~~IKKε~~ and suppresses innate immunity. These findings suggest that the VP2 protein of picornaviruses functions as a potent antagonist of innate immunity by targeting the type I IFN signaling pathway, which will provide valuable insights for development of picornaviral vaccines and antiviral/antitumor therapeutics.

Results

SVA VP2 protein decreased type I IFN production and ISGs expression

Type I IFN pathway is a pivotal regulator of the host antiviral response. SVA non-structural proteins are often known as the key modulators of the host antiviral defenses. However, the functions of SVA structural proteins in modulating host innate immunity during the early stages of infection remain incompletely understood and warrant further investigation. To investigate the potential role of SVA structural proteins in regulating innate immune response, we examined their effects on Sendai virus (SeV)-induced *IFN*~~*-β*~~*B* and *ISRE* (interferon-stimulated response element) promoters activation. The results revealed that the SVA VP2 protein significantly inhibited the activation of both the *IFN*~~*-β*~~*B* and *ISRE* promoters (**Figure 1A and 1B**). The successful expression of SVA structural proteins was confirmed by western blotting (**Fig. S1A and S1B**). Furthermore, overexpression of the SVA VP2 protein dose-dependently suppressed the activation of the *IFNB/IFN-β* promoter triggered by SeV and p~~P~~oly(I:C) (**Figure 1C and 1D**). The successful expression of SVA VP2 protein was also verified by western blotting analysis (**Fig. S1C and S1D**). Additionally, the mRNA expression levels of *IFNB*, *IFIT2/ISG54*, *IFIT1/ISG56* and

OAS1, which were induced by SeV and pPoly(I:C) in HEK-293T cells, were significantly reduced in the presence of the SVA VP2 protein (**Figure 1E and 1F**). Similarly, the upregulation of *IFNB, IFIT2, IFIT1-β, ISG15, ISG54* and *OAS1* mRNA levels induced by SeV and pPoly(I:C) in PK-15 cells was markedly repressed by VP2 (**Fig. S1E and S1F**). In addition, we examined the effect of knockdown of VP2 on the expression of type I IFN and ISGs during SVA infection. The results showed that SVA infection could significantly down-regulate the expression of *IFN-βB*, *ISG54IFIT2*, *ISG56IFIT1* and *OAS1* induced by pPoly(I:C), but this inhibitory effect was weakened in VP2 knockdown cells (**Figure 1G**). These findings demonstrate that SVA VP2 effectively blocks type I IFN production and ISGs expression, thereby attenuating the host antiviral response.

SVA VP2 inhibited the expression of IKBKEIKKε

To determine whether the VP2 protein negatively regulates type I IFN signaling by targeting components of the RIG-IRIGI-like receptor (RLR) pathway, we assessed the expression levels of key RLR pathway components, including IFIH1/MDA5, RIGI, MAVS, TRAF3, TRAF6, TBK1, IKBNKE, IRF3, and IRF7, in the presence or absence of the SVA VP2 protein. The results showed that VP2 specifically and significantly downregulated the expression of IKBKEIKKε, while having no significant impact on the expression of the other components tested (**Figure 2A**). To further elucidate this effect, we conducted a dose-dependent assay. Overexpression of SVA VP2 was found to reduce the expression of HA-tagged IKBKEIKKε in a dose-dependent manner (**Fig. S2A**). In contrast, the expression of HA-RIG-I (**Fig.

S2B) and HA-IRF3 (**Fig. S2C**) remained unaffected by VP2 overexpression. Furthermore, we examined the effect of SVA VP0 on the expression of IKBKE~~IKKε~~, and found that the presence of VP0 significantly down-regulated the expression of IKBKE~~IKKε~~ in a dose-dependent manner (**Fig. S2D**). To verify that the observed downregulation of IKBKE~~IKKε~~ was mediated by VP2, we transfected cells with plasmids expressing Flag-tagged VP2, and detected the expression of endogenous IKBKE~~IKKε~~. The results showed that the endogenous IKBKE~~IKKε~~ expression was reduced in the presence of VP2 showing a dose-dependent manner (**Figure 2B**), while the expression of ~~RIG-I~~RIGI (**Figure 2C**) and IRF3 (**Figure 2D**) was not impacted. Additionally, our data indicated that SVA VP2 had no significant effect on *IKBKE* mRNA expression (**Figure 2E**), suggesting that the downregulation of IKBKE~~IKKε~~ by VP2 occurs at the protein level rather than through transcriptional regulation**.**

The expression of IKBKE~~IKKε~~ during SVA infection was further investigated. As SVA infection progressed, the protein levels of IKBKE~~IKKε~~ also progressively decreased (**Figure 2F**), the mRNA levels of *IKBKE*~~*IKKε*~~ had no significant effect (**Figure 2G**). We also extended our experiments to porcine PK-15 cells. Similar to the findings in human cells, SVA VP2 dose-dependently degraded endogenous I IKBKE~~KKε~~ at the protein level in PK-15 cells (**Fig. S2E**), while having no effect on ~~IKKε~~*IKBKE* mRNA expression (**Fig. S2F**). Furthermore, the expression of IKBKE~~IKKε~~ was downregulated as SVA infection progressed (**Fig. S2G**), while the transcription of *IKBKE*~~IKKε~~ had no effect in PK-15 cells (**Fig. S2H**). These findings suggest that SVA VP2 specifically targets IKBKE~~IKKε~~ for degradation at the protein

level in both human and porcine cells, thereby inhibiting IFN-mediated signaling.

SVA VP2 protein interacted with ~~RIG-I~~RIGI and IKBKE~~IKKε~~

To elucidate the interactions of SVA VP2 with key proteins involved in the innate immune response, we conducted co-Immunoprecipitation (co-IP) assays after co-transfection of VP2 with MDA5, ~~RIG-I~~RIGI, MAVS, TRAF6, TBK1, IRF3, IRF7, IKBKE~~IKKε~~, TANK, or TRAF3 in HEK-293T cells. The results indicated that VP2 specifically interacted with ~~RIG-I~~RIGI and IKBKE~~IKKε~~ (**Figure 2H**). To further confirm these interactions, HEK-293T cells were transfected with plasmids expressing Flag-VP2 and HA-IKBKE~~IKKε~~ or Flag-VP2 and HA-~~RIG-I~~RIGI. The co-IP experiments were then performed, which validated the specific interactions between VP2 and IKBKE~~IKKε~~, as well as VP2 and ~~RIG-I~~RIGI (**Figure 2I and S2I**). Reverse ~~Co~~co-IP experiments also consistently demonstrated these interactions (**Fig. S2J and S2K**). To verify the interaction between VP2 and IKBKE~~IKKε~~ during viral infection, HEK-293T cells were either mock-infected or infected with SVA for 12 h. Immunoprecipitation with anti-VP2 antibody followed by western blotting analysis confirmed the interaction between VP2 and IKBKE~~IKKε~~ during SVA infection (**Figure 2J**).

Additionally, we examined the subcellular localization of SVA VP2 and IKBKE~~IKKε~~ in the context of viral infection. HEK-293T cells were either mock-infected or infected with SVA for 12 h. A clear colocalization of IKBKE~~IKKε~~ and VP2 was observed in the cytoplasm (**Figure 2K**). To ensure image representativeness, we showed more cells and performed a statistical analysis of VP2

and IKBKE~~IKKε~~ co-localization (**Fig. S2L**). Similarly, the colocalization of ~~RIG-I~~RIGI and SVA VP2 was investigated. HEK-293T cells were transfected with HA-~~RIG-I~~RIGI expressing plasmids and then either mock-infected or infected with SVA. Colocalization of VP2 and ~~RIG-I~~RIGI in the cytoplasm was also observed (**Fig. S2M**).

Given that SVA VP2 protein interacts with IKBKE~~IKKε~~ and ~~RIG-I~~RIGI, we sought to determine whether VP2 affects the signal transduction mediated by these proteins. We investigated the effect of VP2 on the interactions between TBK1-~~IKKε~~IKBKE, IKBKE~~IKKε~~-IRF3, and ~~RIG-I~~RIGI-MAVS. The results showed that the interaction between TBK1 and IKBKE~~IKKε~~ was significantly inhibited in the presence of VP2 (**Figure 2L**), and the binding of IKBKE~~IKKε~~ with IRF3 was also reduced (**Figure 2M**). However, VP2 did not significantly affect the interaction between ~~RIG-I~~RIGI and MAVS (**Fig. S2N**). Furthermore, we examined the effect of VP2 on *~~RIG-I~~RIGI* at the mRNA level and found that VP2 did not affect the transcription of *~~RIG-I~~RIGI* (**Fig. S2O**). Therefore, the functional implications of the interaction between VP2 and ~~RIG-I~~RIGI need further investigation. Our findings suggest that the degradation of IKBKE ~~IKKε~~ by VP2 may impede the signal transduction of the type I IFN pathway.

SVA VP2 promoted the degradation of IKBKE~~IKKε~~ through the autophagy pathway

The ubiquitin-proteasome and autophagy-lysosome systems are the primary mechanisms for protein degradation in eukaryotic cells. To elucidate the degradation mechanism of IKBKE~~IKKε~~ by SVA VP2, we transfected HEK-293T cells with

plasmids expressing Flag-VP2 and treated them with either the proteasome inhibitor MG132 or lysosome inhibitors chloroquine (CQ) and 3-methyladenine (3-MA). The results showed that CQ and 3-MA treatment could reverse the VP2-induced degradation of IKBKE~~IKKε~~, whereas MG132 had no such effect (**Figure 3A**). This suggested that VP2 mediates IKBKE~~IKKε~~ degradation through the autophagy-lysosome pathway.

SVA VP2 induces mitophagy in BHK-21 cells [28]. We observed that the ectopic expression of VP2 prompted the down-regulation of SQSTM1 and significantly increased the endogenous conversion of LC3-I to LC3-II in both HEK-293T cells **(Figure 3B)** and PK-15 cells (**Fig. S3A**). Furthermore, overexpression of VP2 increased the formation of LC3 puncta (**Figure 3C**). To further investigate whether VP2 modulates early or late autophagy, we examined the effect of VP2 on the fluorescence of RFP-GFP-LC3 by IFA, and the results showed that the presence of VP2 induces the formation of autolysosomes (**Fig. S3B**). In addition, VP2 interacted with IKBKE~~IKKε~~ but did not interact with SQSTM1~~P62~~ or LC3, indicating that VP2 induces autophagy rather than directly acting on ~~P62~~SQSTM1 and LC3 (**Fig. S3C**).

Autophagic pathways can be classified into canonical and selective autophagy. ULK1 (unc-51 like autophagy activating kinase 1), ATG13 (autophagy related 13), and BECN1 (beclin 1) play crucial roles in the initiation of the canonical autophagic pathway [29, 30]. We found that VP2 did not interact with these three molecules (**Fig. S3D**), suggesting that VP2 may not function via the canonical autophagic pathway. In the selective autophagic pathway, cargo receptors facilitate the delivery of substrates

to autophagosomes. To clarify the relationship between autophagy receptors and IKBKE~~IKKε~~, we co-transfected autophagy receptor proteins (including NBR1, OPTN, CALCOCO2~~NDP52~~, ~~p62~~SQSTM1, and ~~Tollip~~TOLLIP) with IKBKE~~IKKε~~, and detected the potential interactions between IKBKE~~IKKε~~ and these proteins. ~~Co~~co-IP experiments showed that IKBKE~~IKKε~~ interacted with NBR1 and CALCOCO2~~NDP52~~, but not with OPTN, ~~p62~~SQSTM1, or ~~Tollip~~TOLLIP (**Fig. S3E**). Immunofluorescence assays also showed that IKBKE~~IKKε~~ co-localized with NBR1 and CALCOCO2~~NDP52~~ in the cytoplasm (**Fig. S3F**). To identify the autophagy receptors responsible for IKBKE~~IKKε~~ degradation, we transfected these autophagy receptor proteins into HEK-293T cells respectively, and found that only CALCOCO2~~NDP52~~ potentiated the degradation effect of VP2 on IKBKE~~IKKε~~ (**Figure 3D**). In addition, we transfected autophagy receptor proteins and infected cells with SVA to detect the interaction between cargo receptors and VP2 in SVA-infected cells. The results showed that VP2 specifically interacted with CALCOCO2~~NDP52~~ but not with other cargo receptors in the context of viral infection (**Figure 3E**). The immunofluorescence assays confirmed that VP2 co-localized with CALCOCO2~~NDP52~~ in SVA-infected cells (**Figure 3F**). Therefore, CALCOCO2~~NDP52~~ plays a pivotal role in mediating the degradation of IKBKE~~IKKε~~ by VP2.

To further confirm the key functions of CALCOCO2~~NDP52~~ related to SVA VP2, we generated *CALCOCO2*~~*NDP52*~~ knockout (*CALCOCO2*~~*NDP52*~~$^{-/-}$) HEK-293T cells, and evaluated the effect of VP2 on ~~IKKε~~IKBKE in *CALCOCO2*~~*NDP52*~~$^{-/-}$ cells. The

results showed that the degradation of ~~IKKε~~IKBKE induced by VP2 was remarkably decreased in *CALCOCO2*~~*NDP52*~~$^{-/-}$ HEK-293T cells (**Figure 3G**). Furthermore, we investigated the effect of *CALCOCO2*~~*NDP52*~~ knockout on IKBKE~~IKKε~~ expression during SVA infection. The temporal expression profiles of IKBKE~~IKKε~~ at 0, 3, 6, 9, and 12 hpi with SVA were downregulated as the infection progressed in wild-type (WT) HEK-293T cells, but no significant difference was observed in *CALCOCO2*~~*NDP52*~~$^{-/-}$ HEK-293T cells. We observed that the expression of CALCOCO2~~NDP52~~ was down~~-~~regulated as the infection progressed. To determine whether this downregulation was induced by VP2, we examined the expression of CALCOCO2~~NDP52~~ in the presence of overexpressed SVA VP2 or 3C. The results indicated that VP2 downregulated CALCOCO2~~NDP52~~ in a dose-dependent manner (**Fig. S3G**), while 3C did not have this effect (**Fig. S3H**). Moreover, SVA replication was inhibited in *CALCOCO2*~~*NDP52*~~$^{-/-}$ cells compared with WT HEK-293T cells (**Figure 3H**). To further confirm the effect of ~~NDP52~~CALCOCO2 on SVA replication, we detected SVA replication in *CALCOCO2*~~*NDP52*~~$^{-/-}$ HEK-293T cells and found that viral replication was inhibited at the mRNA level as well (**Figure 3I**). Additionally, cell culture supernatants were collected to measure viral titers, and we found that *CALCOCO2* ~~*NDP52*~~ knockout significantly reduced viral titers (**Figure 3J**). CALCOCO2~~NDP52~~ plays a crucial role in the replication of SVA. To evaluate the effect of CALCOCO2 ~~NDP52~~ on the expression of type I IFN and ISGs during SVA infection, we detected the expression of type I IFN and ISGs in *CALCOCO2*~~*NDP52*~~$^{-/-}$ cells. The results showed that the downregulation of p~~P~~oly(I:C)-triggered ISGs by

SVA was abrogated in *CALCOCO2~~NDP52~~*$^{-/-}$ cells (**Figure 3K**). Collectively, these results suggest that VP2 promotes CALCOCO2~~NDP52~~-mediated selective autophagic degradation of IKBKE ~~IKKε~~ and facilitates SVA replication.

VP2 promoted the degradation of IKBKE ~~IKKε~~ by enhancing its K33-linked ubiquitination at the K490 residue

During selective autophagy, substrates are typically modified by ubiquitin and subsequently recognized by cargo receptors, which deliver them to autophagosomes for degradation [10, 31]. Therefore, we examined whether VP2 could promote the ubiquitination of IKBKE~~IKKε~~ and found that the presence of VP2 significantly induced a dose-dependent increase in IKBKE~~IKKε~~ ubiquitination (**Figure 4A**). Common ubiquitination involves seven types of linkages (K6-, K11-, K27-, K29-, K33-, K48-, and K63-linkages). To identify the specific ubiquitination manner involved in VP2-mediated IKBKE~~IKKε~~ degradation, we performed co-IP experiments and found that only K33-linked ubiquitination of IKBKE~~IKKε~~ was increased in the presence of VP2 (**Fig. S4A**). Additionally, VP2 could promote the endogenous K33-linked ubiquitination of IKBKE~~IKKε~~ (**Figure 4B**), while the endogenous K63-linked ubiquitination was not affected by VP2 overexpression (**Fig. S4B**). These data suggested that VP2 promotes K33-linked ubiquitination of IKBKE~~IKKε~~.

To identify the potential ubiquitinate on sites of IKBKE~~IKKε~~ targeted by VP2, we used the GPS-Uber database to predict the ubiquitination sites of IKBKE~~IKKε~~ (**Table 1**). A systematic lysine (K) to arginine (R) mutation was carried out to detect the ubiquitination sites involved in VP2-mediated IKBKE~~IKKε~~ degradation. The

results showed that mutation of lysine 490 to arginine (K490R) considerably blocked the degradation of IKBKE~~IKKε~~ mediated by VP2, whereas other mutations (K25R, K30R, K61R, K154R, K401R, K416R, K529R, K549R, K578R, K609R, or K677R) did not significantly affect the degradation (**Figure 4C**). To further confirm the significance of K490, we evaluated the effect of VP2 on the ubiquitination of the IKBKE^{K490R} mutant. The results showed that the promotion of total ubiquitination and K33-linked ubiquitination of IKBKE^{K490R}~~IKKε K490R~~ mutant by VP2 was almost completely abolished **(Figure 4D)**. These data suggested that VP2 interacts with IKBKE~~IKKε~~ at the K490 residue and facilitates K33 ubiquitination, leading to the subsequent degradation of IKBKE~~IKKε~~.

RNF114 was the E3 ligase that mediates IKBKE~~IKKε~~ ubiquitination induced by SVA VP2

SVA VP2 promotes the ubiquitination of IKBKE~~IKKε~~, but it lacks intrinsic E3 ubiquitin ligase activity. Therefore, we hypothesized that VP2 might function as a scaffold to link IKBKE~~IKKε~~ to its E3 ligase, thereby facilitating its ubiquitination and subsequent degradation. To elucidate this mechanism, we transfected HEK-293T cells with Flag-VP2 and screened potential E3 ligases (TRIM17, TRIM25, TRIM28, RNF114, TRIP12) from the VP2 interactome using mass spectrometry (MS) analysis (**Fig. S5A**). ~~Co~~co-IP experiments confirmed that VP2 interacted with all the E3 ubiquitin ligases tested (**Fig. S5B**). Further analysis revealed that IKBKE~~IKKε~~ specifically interacted with TRIM28 and RNF114 (**Figure 5A**). Immunofluorescence assays showed that RNF114 and IKBKE~~IKKε~~ co-localized in the cytoplasm, whereas

TRIM28 was predominantly localized in the nucleus (**Figure 5B**). We then examined the effects of RNF114 and TRIM28 on IKBKE~~IKKε~~ expression and found that RNF114 but not TRIM28 could degrade IKBKE~~IKKε~~. Importantly, only RNF114 enhanced the degradation of IKBKE~~IKKε~~ in the presence of VP2 (**Figure 5C**). This suggests that RNF114 may be the key E3 ligase involved in VP2-mediated IKBKE~~IKKε~~ degradation. To test this hypothesis, we used RNA interference to knock down *RNF114* and observed that IKBKE~~IKKε~~ degradation was reversed in *RNF114*-knockdown cells (**Figure 5D**).

Next, we investigated whether RNF114 mediated the ubiquitination of IKBKE~~IKKε~~. As expected, overexpression of RNF114 promoted K33-linked ubiquitination of IKBKE~~IKKε~~ but had no effect on K63-linked ubiquitination (**Fig. S5C**). To determine whether RNF114 mediates other ubiquitination manner of IKBKE~~IKKε~~, we conducted a ~~Co~~co-IP experiment and found that RNF114 also catalyzes the K27 and K48-linked ubiquitination of IKBKE~~IKKε~~ (**Fig. S5D**). Additionally, we found that VP2 could upregulate the expression of RNF114 (**Figure 5E**). Subsequently, the ~~Co~~co-IP experiments further showed that VP2 enhanced the interaction between IKBKE~~IKKε~~ and RNF114 in a dose-dependent manner (**Figure 5F**). Therefore, VP2 promotes the ubiquitination of IKBKE~~IKKε~~ by enhancing the binding of IKBKE~~IKKε~~ and RNF114. Previous studies have shown that RNF114 undergoes auto-ubiquitination, our results showed that VP2 significantly enhanced the expression levels of RNF114. To detect the role of VP2 in stabilizing RNF114, we performed a ~~Co~~co-IP experiment, and the results indicated that the presence of VP2

reduced the ubiquitination of RNF114 (**Fig. S5E**). In addition, we investigated the effect of knockdown of *RNF114* on the expression of IKBKE~~IKKε~~ during SVA infection. In the control (si-NC) HEK-293T cells, with the extension of SVA infection time, the temporal expression profiles of IKBKE~~IKKε~~ at 0, 4, 8 and 12 hpi were downregulated, but no significant difference was observed in the *RNF114* knockdown HEK-293T cells. Furthermore, we found SVA replication in *RNF114* knockdown cells was inhibited (**Figure 5G**). To further confirm the effect of RNF114 on SVA replication, we detected SVA replication in *RNF114* knockdown HEK-293T cells and found that SVA replication was inhibited at mRNA level (**Figure 5H**). Moreover, the viral titer of SVA reduced with *RNF114* decrease (**Figure 5I**). Similarly, we investigated whether RNF114 affects the expression of type I IFN and ISGs induced by p~~P~~oly(I:C) during SVA infection. Our results demonstrated that the downregulation of p~~P~~oly(I:C)-induced ISGs by SVA was significantly attenuated upon RNF114 knockdown (**Figure 5J**). Taken together, these data indicate that VP2 degrades IKBKE~~IKKε~~ by recruiting the E3 ubiquitin ligase RNF114, thereby increasing K33-linked ubiquitination of IKBKE~~IKKε~~ and impairing the host antiviral response, facilitating SVA replication.

The regions aa 1-80 and aa 201-284 of VP2 interacted with IKBKE~~IKKε~~ and mediated its degradation

To identify the regions of VP2 responsible for IKBKE~~IKKε~~ degradation, we generated seven deletion mutants (**Figure 6A**). Our results revealed that deletion of the regions aa 1-40, aa 41-80, aa 201-240, and aa 241-284 abolished the ability of

VP2 to degrade IKBKE~~IKKε~~, whereas other deletion mutants did not affect this degradation (**Figure 6B**). Further pull-down assays confirmed that the regions aa 1-40, aa 41-80, and aa 241-284 were the primary binding regions for VP2 interaction with IKBKE~~IKKε~~. Additionally, deletion of the regions aa 121-160 or aa 201-240 in VP2 reduced the interaction of VP2 with IKBKE~~IKKε~~ (**Figure 6C**). Besides, we examined the effects of these VP2 deletion mutants on the activation of *IFNB~~-β~~* and *ISRE* promoters induced by SeV. The results showed that the inhibitory effects of VP2 mutants (aa 1-40, aa 41-80, aa 201-240, and aa 241-284) on the activation of *IFN~~-β~~B* and *ISRE* promoters were abolished (**Figure 6D and 6E**). Therefore, the regions aa 1-80 and aa 201-284 of VP2 are the key regions responsible for inhibiting the activation of the type I IFN pathway.

To identify the key domains of IKBKE~~IKKε~~ that interact with VP2, we constructed deletion mutants IKBKE~~IKKε~~-ΔPK and IKBKE~~IKKε~~-ΔDDX3X based on the domains of IKBKE~~IKKε~~ (**Figure 6F**). Overexpression of VP2 still induced degradation of both IKBKE~~IKKε~~-ΔPK and IKBKE~~IKKε~~-ΔDDX3X (**Figure 6G**). ~~Co~~co-IP experiments also showed that VP2 interacted with the two domains (**Figure 6H**). Therefore, the regions aa 1-80 and aa 201-284 of VP2 are the key domains for degrading IKBKE~~IKKε~~ and blocking the activation of the type I IFN pathway.

The E148 residue of IKBKE~~IKKε~~ and the C34, D39, T41, and R210 residues of VP2 played crucial roles in IKBKE~~IKKε~~-VP2 interaction

To determine the interaction interfaces and residues mediating the IKBKE~~IKKε~~-VP2 interaction, we utilized the SWISS-MODEL online service for homologous modeling

to construct models of the VP2 and IKBKE~~IKKε~~ proteins. Subsequently, we employed the ZDOCK online service (https://zdock.wenglab.org/) to predict the interactions between VP2 and IKBKE~~IKKε~~. The predictions suggested that IKBKE~~IKKε~~ forms one salt bridge, one attractive charge interaction, and ten hydrogen bonds with VP2. Specifically, E148 of IKBKE~~IKKε~~ forms a salt bridge with R210 of VP2. R709 of IKBKE~~IKKε~~ engage in attractive charge interactions with D39 of VP2. Additionally, Q368, T374, E355, S377, V710, N708, L306, L707, S377, and S303 of IKBKE~~IKKε~~ form hydrogen bonds with E257, Q27, T16, Q27, C34, T41, T253, R263, Q27, and G251 of VP2, respectively (**Figure 7A and Table 2**).

To identify the critical amino acid residues responsible for IKBKE~~IKKε~~-VP2 interaction, we generated a series of mutants for both IKBKE~~IKKε~~ and VP2, and performed ~~Co~~co-IP assays to assess their interactions. WT IKBKE~~IKKε~~ and mutants S303A, L306A, Q368A, T374A, S377A, and L707A exhibited strong interactions with VP2, while mutants E355A, N708A, R709A, and V710A showed reduced binding. Notably, the E148A mutant of IKBKE~~IKKε~~ failed to interact with VP2 (**Figure 7B**). To further explore the role of the E148 residue in the degradation of IKBKE~~IKKε~~ by VP2, we co-transfected cells with VP2 along with either WT IKBKE~~IKKε~~ or the IKBKE^{E148A} mutant. The results showed that the degradation of the IKBKE^{E148A} ~~IKKε E148A~~ mutant by VP2 was markedly diminished compared to that of WT IKBKE~~IKKε~~ (**Figure 7C**). These findings demonstrate that the E148 residue of IKBKE~~IKKε~~ is crucial for its interaction with VP2 and subsequent degradation by this protein.

In ~~Co~~co-IP experiments involving VP2 mutants, WT VP2 and the G251A-T253A mutant were found to co-immunoprecipitate with IKBKE~~IKKε~~. In contrast, mutants T16A, Q27A, E257A, and R263A exhibited significantly weakened interactions with IKBKE~~IKKε~~. Notably, mutants C34A, D39A-T41A and R210A completely failed to interact with IKBKE~~IKKε~~ (**Figure 7D**). In addition, the degradation effects of the VP2 C34A, D39A-T41A and R210 mutants on IKBKE~~IKKε~~ were abolished (**Figure 7E**). Collectively, these data suggest that the interaction between VP2 and IKBKE~~IKKε~~ is mediated by multiple key sites. Specifically, the E148 residue of IKBKE~~IKKε~~ and the C34, D39, T41, and R210 residues of VP2 play particularly critical roles in mediating the interaction and subsequent degradation of ~~IKKε~~IKBKE by VP2.

Picornavirus VP2 proteins broadly degraded IKBKE~~IKKε~~ and inhibited type I IFN signaling pathway

Given that SVA VP2 inhibits type I IFN signaling, we investigated whether VP2 proteins from other picornaviruses share this ability. We constructed expression plasmids for FMDV-VP2 and EV71-VP2 and observed that all these VP2 proteins could suppress SeV-induced *IFN-~~β~~B* and *ISRE* promoter activity (**Figure 8A**). Therefore, picornavirus VP2 proteins broadly inhibit type I IFN signaling.

To explore whether the inhibitory mechanisms of FMDV-VP2 and EV71-VP2 on the IFN pathway are similar to that of SVA-VP2, we examined their effects on IKBKE~~IKKε~~ expression. In HEK-293T cells, overexpression of SVA-VP2 and EV71-VP2 caused downregulation of IKBKE~~IKKε~~, decreased ~~P62~~SQSTM1 levels,

and increased the endogenous conversion of LC3-I to LC3-II, whereas FMDV-VP2 had no such effect (**Figure 8B**). Coco-IP assays showed that SVA-VP2 and EV71-VP2 interacted with IKBKEIKKε in HEK-293T cells, while FMDV-VP2 did not (**Figure 8C**). To further investigate the effect of FMDV-VP2 on IKBKEIKKε, we overexpressed FMDV-VP2 and SVA-VP2 in porcine PK-15 cells (susceptible cell line for both SVA and FMDV) and found that both FMDV-VP2 and SVA-VP2 could inhibit the expression of porcine IKBKEIKKε (**Figure 8D**) and interact with porcine IKBKEIKKε (**Figure 8E**).

To confirm that SVA-VP2, EV71-VP2, and FMDV-VP2 degrade IKBKEIKKε through the autophagy pathway, we overexpressed SVA-VP2 and EV71-VP2 in *ATG7* knockout ($ATG7^{-/-}$) HEK-293T cells and detected IKBKEIKKε expression. The results showed that the downregulation effects of SVA-VP2 and EV71-VP2 on IKBKEIKKε were abolished in $ATG7^{-/-}$ cells (**Figure 8F**). Similar assays conducted in *ATG5* knockout ($ATG5^{-/-}$) PK-15 cells revealed that SVA-VP2 and FMDV-VP2 did not degrade IKBKEIKKε in the absence of ATG5 (**Figure 8G**). Furthermore, we examined whether the degradation of IKBKEIKKε by EV71-VP2 and FMDV-VP2 depended on CALCOCO2NDP52. We detected the degradation of IKBKEIKKε by EV71-VP2 in $CALCOCO2NDP52^{-/-}$ HEK-293T cells. The results showed that the degradation of IKBKEIKKε by EV71-VP2 was partly abolished with *CALCOCO2NDP52* knockout (**Figure 8H**). We also designed the interfering RNA targeting porcine *CALCOCO2NDP52* and found that knockdown of CALCOCO2NDP52 weakened the degradation of IKBKEIKKε by FMDV-VP2 in

PK-15 cells (**Figure 8I**). To determine whether the picornavirus VP2 proteins exhibit analogous mechanisms due to their sequence similarity, we performed a sequence alignment of the VP2 proteins from SVA, FMDV, and EV71. The results revealed that the key residue R210 in SVA VP2, which is critical for its interaction with IKBKE~~IKKε~~, is highly conserved across the VP2 proteins of SVA, FMDV, and EV71 (**Fig. S6**). These results demonstrate that picornavirus VP2 proteins share a conserved function in degrading IKBKE~~IKKε~~ and inhibiting the type I IFN signaling pathway through the autophagy pathway.

Autophagy was crucial in picornavirus replication

To further investigate the role of autophagy in picornavirus replication, we examined the replication of SVA and EV71 in HEK-293T cells with ATG7 knockout. The results showed that the downregulation of IKBKE~~IKKε~~ induced by SVA infection was abolished in *ATG7*$^{-/-}$ cells. The expression of the SVA 3D protein (used as an indicator of viral replication) was barely detectable by western blotting (**Figure 9A**), and the transcription of the SVA 3D gene was significantly downregulated as well (**Fig. S7A**). Additionally, cell culture supernatants were collected to measure viral titers, revealing that SVA replication was significantly reduced (**Figure 9B**). Similarly, in *ATG7*$^{-/-}$ HEK-293T cells, the downregulation of IKBKE~~IKKε~~ induced by EV71 infection was also abolished. The expression of the EV71 3D protein decreased (**Figure 9C**), the transcription of the EV71 3D gene was blocked (**Fig. S7B**), and the viral titers also indicated weakened replication of EV71 (**Figure 9D**). Furthermore, we examined the replication of SVA and FMDV in *ATG5*$^{-/-}$ PK-15 cells. The results showed that the

replication of SVA in *ATG5*$^{-/-}$ PK-15 cells was similar to that in *ATG7*$^{-/-}$ HEK-293T cells (**Figure 9E, 9F and S7C**). FMDV infection induced downregulation of IKBKE~~IKKε~~, but this effect was not observed in *ATG5*$^{-/-}$ PK-15 cells (**Figure 9G**). The expression of the FMDV 3D protein decreased (**Figure 9G**), the transcription of the FMDV 3D gene was inhibited (**Fig. S7D**), and the viral titer of FMDV was significantly reduced (**Figure 9H**).

To further verify the role of CALCOCO2~~NDP52~~ in the replication of EV71 and FMDV, we evaluated the viral infection-induced degradation of IKBKE~~IKKε~~ and viral replication in *CALCOCO2*~~*NDP52*~~$^{-/-}$ HEK-293T cells. The results revealed that the down-regulation of IKBKE~~IKKε~~ induced by EV71 infection was blocked in *CALCOCO2*~~*NDP52*~~$^{-/-}$ cells, and the expression of EV71 3D protein was significantly inhibited (**Figure 9I**). Consistently, the transcription of EV71 3D gene was blocked (**Fig. S7E**), and the viral titer decreased (**Figure 9J**). Furthermore, in *CALCOCO2*~~*NDP52*~~ knocked-down PK-15 cells, the down-regulation of IKBKE~~IKKε~~ induced by FMDV disappeared, the expression of FMDV 3D protein decreased (**Figure 9K**), the transcription of the FMDV 3D gene was blocked (**Fig. S7F**), and the viral titer of FMDV also decreased (**Figure 9L**). Taken together, these results indicate that picornaviruses downregulate IKBKE~~IKKε~~ through the autophagy-lysosome pathway to promote viral replication.

To further investigate the function of VP2 in picornavirus replication, we investigated the effects of VP2 overexpression on viral replication. We overexpressed the SVA-VP2 protein in HEK-293T cells and observed that VP2 significantly

upregulated the expression of SVA 3D at the protein level **(Fig. S7G)**, and the transcription of SVA increased as well **(Fig. S7H)**. To further investigated the effect of FMDV-VP2 and EV71-VP2 on viral replication. We transfected PK-15 cells with FMDV VP2 and found that FMDV-VP2 promoted FMDV replication at both the protein and mRNA levels **(Fig. S7I and S7J)**. Similarly, we transfected HEK-293T cells with EV71-VP2 and then infected them with EV71. The replication of EV71 was assessed using western blotting and qPCR. The results indicated that overexpression of EV71-VP2 significantly enhanced EV71 replication **(Fig. S7K and S7L)**. These findings demonstrate that the VP2 protein of picornaviruses, including SVA, FMDV, and EV71, plays a crucial role in promoting viral replication.

DISCUSSION

The innate immune system serves as the primary defense against viral invasion and plays a crucial role in activating the host antiviral response. SVA has evolved multiple strategies to effectively suppress the host innate immune response. For instance, the viral 3C protein disrupts type I IFN production by cleaving MAVS, TRIF, and TANK [32]. Additionally, 3C blocks IFN~~-β~~B expression by inhibiting the ubiquitination of ~~RIG-I~~RIGI, TBK1, and TRAF3 [33]. It also degrades IRF3 and IRF7, thereby inhibiting the activation of the IFN pathway [34]. SVA 2AB protein forms a large complex with MARCHF8 and MAVS, subsequently degrading these proteins and inhibiting the type I IFN response [25]. Therefore, SVA employs multiple viral proteins and mechanisms to inhibit host innate immunity. Various nonstructural proteins are involved in this process. However, the precise functions of SVA structural

proteins in modulating host innate immunity remain unknown. In this study, we discovered for the first time that the structural protein VP2 of SVA significantly inhibits the activation of SeV-induced *IFN*~~*-β*~~*B* and *ISRE* promoters, as well as the expression of ISGs induced by SeV and poly(I:C), thus blocking the activation of the type I IFN pathway.

The IKBKE~~IKKε~~ protein is a key adapter in antiviral innate immune signaling, phosphorylating IRF3 and promoting the production of type I IFN to combat viral infections [35]. Throughout viral evolution, various viral proteins have targeted IKBKE~~IKKε~~ to evade or suppress host innate immunity. For example, the ZIKV NS5 protein interacts with and degrades IKBKE~~IKKε~~, inhibiting the phosphorylation of IRF3 and suppressing the type I IFN pathway [36]. The ASFV pS273R protein interacts with IKBKE~~IKKε~~ and disrupts its interaction with STING, blocking the cGAS-STING signaling pathway [37]. Dengue Virus 2 NS2B interacts with IKBKE~~IKKε~~ and impairs the ~~RIG-I~~RIGI-directed antiviral response [38]. Hepatitis C virus (HCV) NS5A specifically inhibits IKBKE~~IKKε~~ downstream signaling cascades through its interaction with IKBKE~~IKKε~~ [39]. Our investigation revealed that the SVA VP2 degrades IKBKE~~IKKε~~ and disrupts the formation of the TBK1-IKBKE~~IKKε~~ and IKBKE~~-IKKε~~-IRF3 complexes, thereby disturbing the IFN signaling cascade.

Autophagy is a double-edged sword during viral infection, as viruses often exploit autophagy to promote their own replication [40]. For instance, PRRSV induces autophagy and enhances its replication by preventing the fusion of

autophagosomes and lysosomes [41]. The initiation of hepatitis C virus (HCV) replication requires the participation of autophagy proteins [42]. Dengue virus-2 (DV2) depends on the ATG5 protein to trigger the autophagic process and promote its replication [43]. Knockdown of autophagy-related genes reduces the production of infectious HCV particles [44]. Modulating cellular autophagy via pharmacological agents or knocking down autophagy proteins suppresses the replication of influenza A virus [45]. In the context of SVA replication, autophagy also plays a significant role [46, 47]. SVA 2C induces mitophagy to facilitate self-replication [28]. Additionally, the selective autophagy receptor SQSTM1 inhibits SVA replication by degrading VP1 and VP3 [48]. Our research discovered that the SVA VP2 protein degrades ~~IKKε~~IKBKE through the autophagy pathway, thereby inhibiting the activation of the type I IFN pathway. Mechanistically, the VP2 protein interacts with ~~IKKε~~IKBKE, inducing K33-linked polyubiquitination catalyzed by the E3 ubiquitin ligase RNF114. In this process, the K490 residue mediates the ubiquitination of ~~IKKε~~IKBKE. Subsequently, the ubiquitinated ~~IKKε~~IKBKE is recognized by ~~NDP52~~CALCOCO2 and delivered to autophagosomes for degradation.

Based on our data, we propose a working model in which the SVA VP2 protein regulates ~~IKKε~~IKBKE and inhibits the type I IFN signaling pathway to promote viral replication (**Figure 10**). Upon SVA infection, RLR-mediated signal transduction is activated. To counteract the host antiviral immune response, the SVA VP2 protein catalyzes the K33-linked ubiquitination of ~~IKKε~~IKBKE, mediated by the E3 ligase RNF114. Subsequently, the VP2 protein recruits the selective autophagy receptor

~~NDP52~~CALCOCO2, which recognizes the ubiquitinated ~~IKKε~~IKBKE and delivers it to autophagosomes for degradation. Similarly, the VP2 proteins of FMDV and EV71 also utilize the selective autophagy receptor ~~NDP52~~CALCOCO2 to transport ~~IKKε~~IKBKE to autophagosomes for degradation. Thus, the ~~IKKε~~IKBKE-mediated innate signaling pathway is disrupted, the activation of the type I IFN pathway is blocked, and viral replication is facilitated. Importantly, we found that picornavirus VP2 proteins share a conserved function in degrading ~~IKKε~~IKBKE and inhibiting the type I IFN signaling pathway through the autophagy pathway.

In summary, our findings provide a novel regulatory mechanism by which autophagy and innate immunity mutually regulate type I IFN signaling during SVA replication. Meanwhile, this mechanism is also present in other picornaviruses. These data provide a new theoretical basis and ideas for the development of antiviral drugs and vaccines against picornaviruses.

Materials and methods

Cells, viruses, and infection

HEK-293T cells (ATCC, CRL-11268), HeLa cells (ATCC, CCL-2), PK-15 cells (ATCC, CCL-33), IBRS-2 cells (ATCC, CRL-1746) and BHK-21 cells (ATCC, CCL-10) were cultured in Dulbecco modified Eagle medium (DMEM; VivaCell, C3110-0500) supplemented with 10% heat-inactivated fetal bovine serum (Excell, FSP500) and maintained at 37 ℃ with 5% CO_2. *CALCOCO2~~NDP52~~*$^{-/-}$, *ATG7*$^{-/-}$ HEK-293T cells were a gift from S~~s~~huai X~~x~~u (Lanzhou Veterinary Research Institute). *ATG5*$^{-/-}$ PK-15 cells were stored in our laboratory [19]. For *CALCOCO2~~NDP52~~*$^{-/-}$

cells the target sequences GGATCACTGTCATTTCTCTC were cloned into pLentiCRISPRv2 (Solarbio, VT000068~~company, catalog number~~), then transfected into HEK-293T cells and purified by puromycin selection. The target sequences of human *ATG7* and porcine *ATG5* were inserted into the pLentiCRISPR plasmid respectively with the puromycin selection gene. All knockout cell lines were verified by western blotting analysis. SVA CH-FJ-2017 strain (GenBank accession number KY747510), previously isolated and preserved in our lab, was utilized for viral infection [49]. The virus propagation and titration were carried out using IBRS-2 cells. The FMDV type O strain O/BY/CHA/2010 was conducted using BHK-21 cells [50]. The EV71 strain H (VR-1432) stored in our laboratory, propagated and titrated using HeLa cells. The 50% tissue culture infectious dose ($TCID_{50}$) was determined using the Reed and Muench method [51]. All virus strains were cryopreserved at −80°C. The viral infection experiments were conducted in accordance with the procedures described in previous studies [52].

Plasmids and antibodies

The indicated SVA gene cDNA and the VP2 gene cDNAs of FMDV, and EV71 were individually cloned into the p3xFLAG-CMV-7.1 vector (Invitrogen, E7533~~, catalog number~~) to generate plasmids expressing Flag-tagged viral proteins. Mammalian expression plasmids for HA-tagged ~~RIG-I~~RIGI, MDA5, MAVS, TRAF3, TRAF6, TANK, ~~IKKε~~IKBKE, TBK1, IRF3 and IRF7, and the *IFN~~-β~~B* and *ISRE* promoter luciferase reporter plasmids, were kindly provided by Professor Hongbing Shu (Wuhan University, China). Flag-tagged NBR1, OPTN, CALCOCO2~~NDP52~~,

~~P62~~SQSTM1, ~~Tollip~~TOLLIP were kindly provided by Shuai Xu (Lanzhou Veterinary Research Institute). *TRIM17*, *TRIM25*, *TRIM28*, *RNF114*, and *TRIP12* cDNA were cloned into pCMV6 vector (Miaoling Biotechnology, P58982~~company, catalog number~~) to generate MYC-Flag-tagged expressing plasmids. A series of Flag-tagged truncated and site-specific mutants of VP2, and HA-tagged truncated and site-specific mutants of ~~IKKε~~IKBKE plasmids were generated by site-directed mutagenesis PCR. All constructed plasmids were analyzed and verified by DNA sequencing.

The commercial antibodies used in this study include: anti-Flag mouse Ab (Sigma, F1804), anti-M~~yc~~YC mouse Ab (Sigma, M5546), anti-HA mouse Ab (Proteintech, 66006-2-Ig), anti-RIGI rabbit Ab (Cell Signaling Technology, 3743S), anti-IFIH1/MDA5 rabbit Ab (Abcam, Ab126630), anti-MAVS rabbit Ab (Cell Signaling Technology, 3993S), anti-TRAF3 rabbit Ab (Cell Signaling Technology, 4729T), anti-TRAF6 rabbit Ab (Cell Signaling Technology, 67591S), anti-TBK1 rabbit Ab (Cell Signaling Technology, 38066S), anti-IRF3 rabbit Ab (Proteintech, 11312-1-AP), anti-IRF7 rabbit Ab (Abcam, ab109255), anti-LC3 rabbit Ab (Proteintech, 14600-1-AP), anti-TUBB/β-tubulin mouse Ab (Abclonal, A12289), anti-IKBKE/IKKε rabbit Ab (Abclonal, A3463), anti-K33 rabbit Ab (Abclonal, A18199), anti-RNF114 rabbit Ab (Abclonal, A10636), anti-CALCOCO2/NDP52 rabbit Ab (Abclonal, A24021), anti-SQSTM1/p62 rabbit Ab (Abclonal, A19700). Anti-VP2 rabbit polyclonal Ab and Anti-3D rabbit polyclonal Ab were prepared by our laboratory previously (unpublished data). Goat anti-mouse (BF03001) or rabbit (BF03008) IgG (H+L) secondary antibodies were purchased from Biodragon

company.

Transfection and reporter Assays

HEK-293T cells grown in 48-well plates were transfected with a combination of 50 ng of reporter plasmid (*IFN-βB*, *ISRE*), 5 ng of pRL-TK (Promega, E2241 catalog number) serving as an internal control, and 50 ng of the specified plasmid. At 24 h post transfection (hpt), the cells were stimulated with SeV for another 16 h, the whole-cell extracts were prepared to assess dual-luciferase activities. The experiment was conducted three times independently.

RNA extraction and real-time PCR

Total RNA was extracted from the cell cultures using TRIzol Reagent (Vazyme, R401-01), and then reverse-transcribed into cDNA with HiScript II Q RT SuperMix for qPCR (Vazyme, R222-01) according to the manufacturer's instructions. The relative amounts of cDNAs were quantified and using ChamQ Universal SYBR qPCR Master Mix (Vazyme, Q711-03) following the manufacturer's protocol to analyze the target genes. The *GAPDH* (glyceraldehyde-3-phosphate dehydrogenase) gene was used as an internal control, and the relative mRNA levels were calculated using $2^{-\Delta\Delta CT}$ method. The qPCR primers used in this study were shown in Table 3.

RNA interference (RNAi)

The siRNA was transfected with jetPRIME DNA transfection reagent (Polyplus Transfection, 101000046company, catalog number) provided into HEK-293T cells and PK-15 cells. The RNF114 and CALCOCO2NDP52 target sequence was purchased from (Sangon Biotech, catalog numbersRNA2508991). The siRNA

sequences are shown in Table 3.

Coimmunoprecipitation and western blotting analysis

HEK-293T cells were cultured in 10-cm dishes, and the monolayer cells were co-transfected with various plasmids. The collected cells were then lysed by radio-immunoprecipitation assay/RIPA lysis buffer and immunoprecipitated with BeyoMag™ Anti-~~Myc~~ MYC Magnetic Beads (Beyotime, P2118), BeyoMag™ Anti-HA Magnetic Beads (Beyotime, P2121) or BeyoMag™ Anti-Flag Magnetic Beads (Beyotime, P2115), then the cells supernatant were incubated with beads at 4° C for 4 h. The precipitated proteins were analyzed by western blot with 10% or 12% SDS-PAGE and transferred onto the nitrocellulose membranes (Cytiva, 10600001~~, catalog number~~). The membrane was blocked by 5% nonfat dried milk diluted in TBST (Yamay, PS103~~company, catalog number~~) with ~~X~~0.5% Tween-20 (Sigma, P1379~~company, catalog number~~) and incubated with appropriate primary antibodies and secondary antibodies, and the antibody-antigen complexes were subsequently visualized using ECL detection reagents (Thermo Fisher Scientific, K-12045-D50~~, catalog number~~).

Confocal immunofluorescence assay

Cells were seeded into glass bottom cell culture dishes and transfected with various plasmids using Lipofectamine 2000 (Thermo Scientific, 11668-019). At 24 hpt, the cells were infected with SVA for 10 h. The cells were then fixed, permeabilized and blocked as previously described [53]. The fixed cells were incubated with primary antibodies overnight, then incubated with secondary antibodies conjugated to Alexa

Fluor™ 488 (Thermo Scientific, A-11001) or Alexa Fluor™ 594 (Thermo Scientific, A-11012) at room temperature for 2 h, cell nuclei were stained with DAPI for 10 min. The cells were visualized using a Leica SP2 confocal microscopy system (Leica Microsystems, Wizla, Germany).

Statistical analysis

All Statistical analysis was performed using GraphPad Prism software version 8.0. A two-tailed Student's *t* test and one-way ANOVA were employed to assess the significance of the data. The statistical significance was indicated in the figures (*$P < 0.05$, **$P < 0.01$, ***$P < 0.001$, ****$P < 0.0001$, ns indicated not significant).

Acknowledgments

This work was supported by grants from the National Natural Science Foundation of China (32273026 to Zixiang Zhu), the open competition program of top ten critical priorities of Agricultural Science and Technology Innovation for the 14th Five-Year Plan of Guangdong Province（2024KJ14 to Haixue Zheng), the Fundamental Research Funds for the Central Universities, the Scientific Funds of Gansu Province (23JRRA1515, 25JRRA1087 and 24CXNA055 to Jijun He, and 24JRRA016 to Xiangle Zhang, 24JRRA804 to Zixiang Zhu, 24JRRA813 to Guoliang Zhu), the Project of National Center of Technology Innovation for Pigs (NCTIP-XD/C03 to Haixue Zheng), the Innovation Project for Young Scientist in Lanzhou (2023-QN-75 to Xiangle Zhang), the Basic Research Fund of LVRI in CAAS (LVRI-1610312022006 to Xiangle Zhang), the Earmarked Fund for CARS-35 and

CARS-39-13 (to Haixue Zheng), and the Fundamental Research Funds for Innovation Team of Gansu Province (23JRRA546 to Zixiang Zhu).

Disclosure statement

The authors declare that they have no conflicts of interest.

Data availability statement

Data are available on reasonable request.

References

1. Armson B, Walsh C, Morant N, et al. The development of two field-ready reverse transcription loop-mediated isothermal amplification assays for the rapid detection of Seneca Valley virus 1. Transboundary and emerging diseases. 2019 Jan;66(1):497-504. doi: 10.1111/tbed.13051. PubMed PMID: 30372584; PubMed Central PMCID: PMCPMC6434928. eng.
2. McKnight KL, Lemon SM. Virology: Ins and outs of picornaviruses. Nature. 2017 Jan 19;541(7637):299-300. doi: 10.1038/nature21116. PubMed PMID: 28102264; eng.
3. Luo D, Wang H, Wang Q, et al. Senecavirus A as an Oncolytic Virus: Prospects, Challenges and Development Directions. Frontiers in oncology. 2022;12:839536. doi: 10.3389/fonc.2022.839536. PubMed PMID: 35371972; PubMed Central PMCID: PMCPMC8968071. eng.
4. Schijven J, Brizee S, Teunis P, et al. Quantitative Assessment of the Health Risk for Livestock When Animal Viruses Are Applied in Human Oncolytic Therapy: A Case Study for Seneca Valley Virus. Risk analysis : an official publication of the Society for Risk Analysis. 2019 May;39(5):982-991. doi: 10.1111/risa.13227. PubMed PMID: 30395685; eng.
5. Segalés J, Barcellos D, Alfieri A, et al. Senecavirus A. Veterinary pathology. 2017 Jan;54(1):11-21. doi: 10.1177/0300985816653990. PubMed PMID: 27371541; eng.
6. Wang Q, Wang J, Zhang L, et al. Substitutions of stem-loop subdomains in internal ribosome entry site of Senecavirus A: Impacts on rescue of sequence-modifying viruses. Journal of Integrative Agriculture. 2024 2024/07/01/;23(7):2391-2406. doi: https://doi.org/10.1016/j.jia.2024.04.019; eng.
7. Hales LM, Knowles NJ, Reddy PS, et al. Complete genome sequence analysis of Seneca Valley virus-001, a novel oncolytic picornavirus. The Journal of general virology. 2008 May;89(Pt 5):1265-75. doi: 10.1099/vir.0.83570-0. PubMed PMID: 18420805.

8. Li K, Wang C, Yang F, et al. Virus-Host Interactions in Foot-and-Mouth Disease Virus Infection. Frontiers in immunology. 2021;12:571509. doi: 10.3389/fimmu.2021.571509. PubMed PMID: 33717061; PubMed Central PMCID: PMCPMC7952751.
9. Ma Y, Galluzzi L, Zitvogel L, et al. Autophagy and cellular immune responses. Immunity. 2013 Aug 22;39(2):211-27. doi: 10.1016/j.immuni.2013.07.017. PubMed PMID: 23973220; eng.
10. Shaid S, Brandts CH, Serve H, et al. Ubiquitination and selective autophagy. Cell death and differentiation. 2013 Jan;20(1):21-30. doi: 10.1038/cdd.2012.72. PubMed PMID: 22722335; PubMed Central PMCID: PMCPMC3524631. eng.
11. Ji CH, Kwon YT. Crosstalk and Interplay between the Ubiquitin-Proteasome System and Autophagy. Molecules and cells. 2017 Jul 31;40(7):441-449. doi: 10.14348/molcells.2017.0115. PubMed PMID: 28743182; PubMed Central PMCID: PMCPMC5547213. eng.
12. Stolz A, Ernst A, Dikic I. Cargo recognition and trafficking in selective autophagy. Nature cell biology. 2014 Jun;16(6):495-501. doi: 10.1038/ncb2979. PubMed PMID: 24875736; eng.
13. Vargas JNS, Hamasaki M, Kawabata T, et al. The mechanisms and roles of selective autophagy in mammals. Nature reviews Molecular cell biology. 2023 Mar;24(3):167-185. doi: 10.1038/s41580-022-00542-2. PubMed PMID: 36302887; eng.
14. Viret C, Duclaux-Loras R, Nancey S, et al. Selective Autophagy Receptors in Antiviral Defense. Trends in microbiology. 2021 Sep;29(9):798-810. doi: 10.1016/j.tim.2021.02.006. PubMed PMID: 33678557; eng.
15. Sui C, Xiao T, Zhang S, et al. SARS-CoV-2 NSP13 Inhibits Type I IFN Production by Degradation of TBK1 via p62-Dependent Selective Autophagy. Journal of immunology (Baltimore, Md : 1950). 2022 Feb 1;208(3):753-761. doi: 10.4049/jimmunol.2100684. PubMed PMID: 34996837; eng.
16. Deng T, Hu B, Wang X, et al. TRAF6 autophagic degradation by avibirnavirus VP3 inhibits antiviral innate immunity via blocking NFKB/NF-κB activation. Autophagy. 2022 Dec;18(12):2781-2798. doi: 10.1080/15548627.2022.2047384. PubMed PMID: 35266845; PubMed Central PMCID: PMCPMC9673932. eng.
17. Zeng Y, Xu S, Wei Y, et al. The PB1 protein of influenza A virus inhibits the innate immune response by targeting MAVS for NBR1-mediated selective autophagic degradation. PLoS pathogens. 2021 Feb;17(2):e1009300. doi: 10.1371/journal.ppat.1009300. PubMed PMID: 33577621; PubMed Central PMCID: PMCPMC7880438. eng.
18. Ma Z, Bai J, Jiang C, et al. Tegument protein UL21 of alpha-herpesvirus inhibits the innate immunity by triggering CGAS degradation through TOLLIP-mediated selective autophagy. Autophagy. 2023 May;19(5):1512-1532. doi: 10.1080/15548627.2022.2139921. PubMed PMID: 36343628; PubMed Central PMCID: PMCPMC10241001. eng.
19. Liu H, Xue Q, Yang F, et al. Foot-and-mouth disease virus VP1 degrades YTHDF2 through autophagy to regulate IRF3 activity for viral replication. Autophagy. 2024 Jul;20(7):1597-1615. doi: 10.1080/15548627.2024.2330105. PubMed PMID: 38516932; PubMed Central PMCID: PMCPMC11210904. eng.
20. Mao R, Zhu Z, Yang F, et al. Picornavirus VP3 protein induces autophagy through the TP53-BAD-BAX axis to promote viral replication. Autophagy. 2024 Sep;20(9):1928-1947. doi: 10.1080/15548627.2024.2350270. PubMed PMID: 38752369; PubMed Central PMCID:

PMCPMC11346532. eng.
21. Zhang H, Wang X, Qu M, et al. Foot-and-mouth disease virus structural protein VP3 interacts with HDAC8 and promotes its autophagic degradation to facilitate viral replication. Autophagy. 2023 Nov;19(11):2869-2883. doi: 10.1080/15548627.2023.2233847. PubMed PMID: 37408174; PubMed Central PMCID: PMCPMC10549200. eng.
22. Sun P, Zhang S, Qin X, et al. Foot-and-mouth disease virus capsid protein VP2 activates the cellular EIF2S1-ATF4 pathway and induces autophagy via HSPB1. Autophagy. 2018;14(2):336-346. doi: 10.1080/15548627.2017.1405187. PubMed PMID: 29166823; PubMed Central PMCID: PMCPMC5902195. eng.
23. Xiang Q, Wan P, Yang G, et al. Beclin1 Binds to Enterovirus 71 3D Protein to Promote the Virus Replication. Viruses. 2020 Jul 14;12(7). doi: 10.3390/v12070756. PubMed PMID: 32674313; PubMed Central PMCID: PMCPMC7411969. eng.
24. Liu ZW, Zhuang ZC, Chen R, et al. Enterovirus 71 VP1 Protein Regulates Viral Replication in SH-SY5Y Cells via the mTOR Autophagy Signaling Pathway. Viruses. 2019 Dec 20;12(1). doi: 10.3390/v12010011. PubMed PMID: 31861844; PubMed Central PMCID: PMCPMC7019657. eng.
25. Sun D, Kong N, Dong S, et al. 2AB protein of Senecavirus A antagonizes selective autophagy and type I interferon production by degrading LC3 and MARCHF8. Autophagy. 2022 Aug;18(8):1969-1981. doi: 10.1080/15548627.2021.2015740. PubMed PMID: 34964697; PubMed Central PMCID: PMCPMC9450971. eng.
26. Song J, Guo Y, Wang D, et al. Seneca Valley virus 3C protease cleaves OPTN (optineurin) to Impair selective autophagy and type I interferon signaling. Autophagy. 2024 Mar;20(3):614-628. doi: 10.1080/15548627.2023.2277108. PubMed PMID: 37930946; PubMed Central PMCID: PMCPMC10936645. eng.
27. Shi Y, Wu Z, Zeng P, et al. Seneca valley virus 3C protease blocks EphA2-Mediated mTOR activation to facilitate viral replication. Microbial pathogenesis. 2024 Jun;191:106673. doi: 10.1016/j.micpath.2024.106673. PubMed PMID: 38705218; eng.
28. Chen M, Zhang X, Kong F, et al. Senecavirus A induces mitophagy to promote self-replication through direct interaction of 2C protein with K27-linked ubiquitinated TUFM catalyzed by RNF185. Autophagy. 2024 Jun;20(6):1286-1313. doi: 10.1080/15548627.2023.2293442. PubMed PMID: 38084826; PubMed Central PMCID: PMCPMC11210902. eng.
29. Boyle KB, Randow F. The role of 'eat-me' signals and autophagy cargo receptors in innate immunity. Current opinion in microbiology. 2013 Jun;16(3):339-48. doi: 10.1016/j.mib.2013.03.010. PubMed PMID: 23623150; eng.
30. Yu L, Chen Y, Tooze SA. Autophagy pathway: Cellular and molecular mechanisms. Autophagy. 2018;14(2):207-215. doi: 10.1080/15548627.2017.1378838. PubMed PMID: 28933638; PubMed Central PMCID: PMCPMC5902171. eng.
31. Cohen-Kaplan V, Livneh I, Avni N, et al. The ubiquitin-proteasome system and autophagy: Coordinated and independent activities. The international journal of biochemistry & cell biology. 2016 Oct;79:403-418. doi: 10.1016/j.biocel.2016.07.019. PubMed PMID: 27448843; eng.
32. Qian S, Fan W, Liu T, et al. Seneca Valley Virus Suppresses Host Type I Interferon Production by Targeting Adaptor Proteins MAVS, TRIF, and TANK for Cleavage. Journal of virology. 2017 Aug 15;91(16). doi: 10.1128/JVI.00823-17. PubMed PMID: 28566380; PubMed Central

PMCID: PMC5533933.

33. Xue Q, Liu H, Zhu Z, et al. Seneca Valley Virus 3C protease negatively regulates the type I interferon pathway by acting as a viral deubiquitinase. Antiviral research. 2018 Dec;160:183-189. doi: 10.1016/j.antiviral.2018.10.028. PubMed PMID: 30408499; PubMed Central PMCID: PMCPMC7111287. eng.

34. Xue Q, Liu H, Zhu Z, et al. Seneca Valley Virus 3C(pro) abrogates the IRF3- and IRF7-mediated innate immune response by degrading IRF3 and IRF7. Virology. 2018 Feb 7;518:1-7. doi: 10.1016/j.virol.2018.01.028. PubMed PMID: 29427864.

35. Fitzgerald KA, McWhirter SM, Faia KL, et al. IKKepsilon and TBK1 are essential components of the IRF3 signaling pathway. Nature immunology. 2003 May;4(5):491-6. doi: 10.1038/ni921. PubMed PMID: 12692549.

36. Lundberg R, Melén K, Westenius V, et al. Zika Virus Non-Structural Protein NS5 Inhibits the RIG-I Pathway and Interferon Lambda 1 Promoter Activation by Targeting IKK Epsilon. Viruses. 2019 Nov 4;11(11). doi: 10.3390/v11111024. PubMed PMID: 31690057; PubMed Central PMCID: PMCPMC6893776. eng.

37. Luo J, Zhang J, Ni J, et al. The African swine fever virus protease pS273R inhibits DNA sensing cGAS-STING pathway by targeting IKKε. Virulence. 2022 Dec;13(1):740-756. doi: 10.1080/21505594.2022.2065962. PubMed PMID: 35437104; PubMed Central PMCID: PMCPMC9067533. eng.

38. Nie Y, Deng D, Mou L, et al. Dengue Virus 2 NS2B Targets MAVS and IKKε to Evade the Antiviral Innate Immune Response. Journal of microbiology and biotechnology. 2023 May 28;33(5):600-606. doi: 10.4014/jmb.2210.10006. PubMed PMID: 36788451; PubMed Central PMCID: PMCPMC10236164. eng.

39. Kang SM, Park JY, Han HJ, et al. Hepatitis C Virus Nonstructural Protein 5A Interacts with Immunomodulatory Kinase IKKε to Negatively Regulate Innate Antiviral Immunity. Molecules and cells. 2022 Oct 31;45(10):702-717. doi: 10.14348/molcells.2022.0018. PubMed PMID: 35993162; PubMed Central PMCID: PMCPMC9589372. eng.

40. Choi Y, Bowman JW, Jung JU. Autophagy during viral infection - a double-edged sword. Nature reviews Microbiology. 2018 Jun;16(6):341-354. doi: 10.1038/s41579-018-0003-6. PubMed PMID: 29556036; PubMed Central PMCID: PMCPMC6907743. eng.

41. Sun MX, Huang L, Wang R, et al. Porcine reproductive and respiratory syndrome virus induces autophagy to promote virus replication. Autophagy. 2012 Oct;8(10):1434-47. doi: 10.4161/auto.21159. PubMed PMID: 22739997; eng.

42. Dreux M, Gastaminza P, Wieland SF, et al. The autophagy machinery is required to initiate hepatitis C virus replication. Proceedings of the National Academy of Sciences of the United States of America. 2009 Aug 18;106(33):14046-51. doi: 10.1073/pnas.0907344106. PubMed PMID: 19666601; PubMed Central PMCID: PMCPMC2729017. eng.

43. Lee YR, Lei HY, Liu MT, et al. Autophagic machinery activated by dengue virus enhances virus replication. Virology. 2008 May 10;374(2):240-8. doi: 10.1016/j.virol.2008.02.016. PubMed PMID: 18353420; PubMed Central PMCID: PMCPMC7103294. eng.

44. Tanida I, Fukasawa M, Ueno T, et al. Knockdown of autophagy-related gene decreases the production of infectious hepatitis C virus particles. Autophagy. 2009 Oct;5(7):937-45. doi: 10.4161/auto.5.7.9243. PubMed PMID: 19625776; eng.

45. Zhou Z, Jiang X, Liu D, et al. Autophagy is involved in influenza A virus replication.

Autophagy. 2009 Apr;5(3):321-8. doi: 10.4161/auto.5.3.7406. PubMed PMID: 19066474; eng.
46. Song J, Hou L, Quan R, et al. Synergetic Contributions of Viral VP1, VP3, and 3C to Activation of the AKT-AMPK-MAPK-MTOR Signaling Pathway for Seneca Valley Virus-Induced Autophagy. Journal of virology. 2022 Jan 26;96(2):e0155021. doi: 10.1128/jvi.01550-21. PubMed PMID: 34757844; PubMed Central PMCID: PMCPMC8791279. eng.
47. Xie SJ, Yan JY, Jiang B, et al. Immune evasion strategies of Seneca Valley virus: mechanisms of host innate immune suppression. Agr Commun-Prc. 2025 Sep;3(3). doi: ARTN 10010010.1016/j.agrcom.2025.100100. PubMed PMID: WOS:001566381800001. eng.
48. Wen W, Li X, Yin M, et al. Selective autophagy receptor SQSTM1/ p62 inhibits Seneca Valley virus replication by targeting viral VP1 and VP3. Autophagy. 2021 Nov;17(11):3763-3775. doi: 10.1080/15548627.2021.1897223. PubMed PMID: 33719859; PubMed Central PMCID: PMCPMC8632295. eng.
49. Zhu Z, Yang F, Chen P, et al. Emergence of novel Seneca Valley virus strains in China, 2017. Transboundary and emerging diseases. 2017 Aug;64(4):1024-1029. doi: 10.1111/tbed.12662. PubMed PMID: 28544501; eng.
50. Liu H, Xue Q, Zhu Z, et al. Foot-and-Mouth Disease Virus Inhibits RIP2 Protein Expression to Promote Viral Replication. Virologica Sinica. 2021 Aug;36(4):608-622. doi: 10.1007/s12250-020-00322-2. PubMed PMID: 33400090; PubMed Central PMCID: PMCPMC8379319. eng.
51. Liu H, Zhu Z, Xue Q, et al. Innate sensing of picornavirus infection involves cGAS-STING-mediated antiviral responses triggered by mitochondrial DNA release. PLoS pathogens. 2023 Feb;19(2):e1011132. doi: 10.1371/journal.ppat.1011132. PubMed PMID: 36745686; PubMed Central PMCID: PMCPMC9934381. eng.
52. Lei CQ, Zhong B, Zhang Y, et al. Glycogen synthase kinase 3β regulates IRF3 transcription factor-mediated antiviral response via activation of the kinase TBK1. Immunity. 2010 Dec 14;33(6):878-89. doi: 10.1016/j.immuni.2010.11.021. PubMed PMID: 21145761; eng.
53. Zhu Z, Wang G, Yang F, et al. Foot-and-Mouth Disease Virus Viroporin 2B Antagonizes RIG-I-Mediated Antiviral Effects by Inhibition of Its Protein Expression. Journal of virology. 2016 Dec 15;90(24):11106-11121. doi: 10.1128/jvi.01310-16. PubMed PMID: 27707918; PubMed Central PMCID: PMCPMC5126369. eng.

Figure legends

Figure 1. SVA VP2 protein inhibits type I IFN signaling pathway. (**A, B**) HEK-293T cells were co-transfected with *IFNB* (50 ng) or *ISRE* (50 ng) luciferase reporter plasmids, along with an internal control plasmid PRL-TK (5 ng) and either an empty vector (Vec) or the indicated viral protein-expressing plasmids (50 ng) for 24 h. The cells were then treated with SeV to activate the promoter *IFNB~~-β~~* (**A**) or *ISRE* promoter (**B**) for an additional 16 h. The luciferase activity was measured by dual luciferase assay. (**C, D**) The *IFN~~-β~~B* luciferase reporter plasmid, PRL-TK internal control plasmid and increasing amounts of Flag-VP2 expressing plasmids (0, 0, 20, 50, or 100 ng) were co-transfected into HEK-293T cells. At 24 h post-transfection (hpt), the cells were treated with SeV (**C**) or p~~P~~oly(I:C) (**D**) for another 16 h. (**E, F**) The mRNA levels of *IFN~~-β~~B*, *~~ISG54~~IFIT2*, *~~ISG56~~IFIT1*, and *OAS1* in HEK-293T cells transfected with either Vec, Flag-VP2 or Flag-VP4 plasmids followed by treatment with SeV (**E**) or p~~P~~oly(I:C) (**F**) were measured by qPCR. (**G**) HEK-293T cells were transfected with NC siRNA or VP2 siRNA for 24 h, followed by transfection with p~~P~~oly(I:C) for another 12 h, then infected with SVA for 12 h. Total RNA was extracted, and the mRNA levels of *IFN~~-β~~B*, *~~ISG54~~IFIT2*, *~~ISG56~~IFIT1*, and *OAS1* were quantified by qPCR. All experiments were repeated three times, with similar results. **P*< 0.05, ***P*< 0.01, ****P*<0.001, *****P*<0.0001, ns, not significant.

Figure 2. SVA VP2 protein inhibits the expression of ~~IKKε~~IKBKE and interacts with it. (**A**) HEK-293T cells were transfected with empty vector or Flag-VP2 plasmids. The cells were lysed at 24 hpt and analyzed by western blotting using the indicated

antibodies. (**B-D**) HEK-293T cells were transfected with 0, 0.25, 0.5 or 1 μg of Flag-VP2 expressing plasmids for 24 h. The expression levels of endogenous ~~IKKε~~IKBKE (**B**), ~~RIG-I~~RIGI (**C**), or IRF3 (**D**) were assessed by western blotting. (**E**) HEK-293T cells were transfected with Flag-VP2 expressing plasmids (0, 0.25, 0.5 or 1 μg) for 24 h. Total RNA was extracted from the cells, and ~~IKKε~~*IKBKE* mRNA levels were quantified by qPCR. (**F, G**) HEK-293T cells were infected with SVA (MOI =0.5) for 0, 4, 8 and 12 h respectively, and the protein expression of ~~IKKε~~IKBKE was analyzed by western blotting (**F**), the mRNA expression of ~~IKKε~~*IKBKE* was detected by qPCR (**G**). (**H**) HEK-293T cells were co-transfected with Flag-VP2 and either Vec, various HA-tagged innate immune molecule-expressing plasmids (MDA5, ~~RIG-I~~RIGI, MAVS, TRAF6, TBK1, IRF3, IRF7, ~~IKKε~~IKBKE, and TANK) or ~~Myc~~MYC-tagged TRAF3. At 36 hpt, the cell lysates were subjected to ~~Co~~co-IP assay analysis. The immunoprecipitated proteins and whole-cell lysates (WCL) were analyzed by western blotting using the specified antibodies. (**I**) HEK-293T cells were co-transfected with HA-~~IKKε~~IKBKE and vector or Flag-VP2 expressing plasmids for 36 h. The cell lysates were immunoprecipitated with anti-HA or control IgG antibodies, and the antigen-antibody complex was subjected to western blotting analysis. (**J**) HEK-293T cells were mock-infected or infected with SVA at an MOI of 0.5 for 12 h. Cell lysates were then immunoprecipitated with anti-VP2 or control IgG antibodies, the antigen-antibody complex were assessed by western blotting. (**K**) HEK-293T cells were mock-infected or infected with SVA (MOI=0.1) for 10 h, after which the colocalization of

~~IKKε~~IKBKE (red) and VP2 (green) was assessed by immunofluorescence assay (IFA). Nuclei were counterstained with DAPI (blue). (**L, M**) HEK-293T cells were co-transfected with increasing amounts of Flag-VP2 and ~~Myc~~MYC-~~IKKε~~IKBKE, along with HA-TBK1 (**L**) or HA-IRF3 (**M**) expressing plasmids. At 24 hpt, the cells were treated with SeV for another 12 h. The cells were lysed and immunoprecipitated with anti-~~Myc~~ MYC antibodies. The immunoprecipitated proteins and WCL were analyzed by western blotting using the specified antibodies.

Figure 3. SVA VP2 promotes the degradation of ~~IKKε~~IKBKE through CALCOCO2~~NDP52~~-mediated selective autophagy. (**A**) HEK-293T cells were co-transfected with empty vector or Flag-VP2. At 24 hpt, the cells were treated with 20 μM MG132, 50 μM chloroquine (CQ), or 10 mM 3-methyladenine (3-MA) for 6 h. The cell lysates were analyzed by western blotting using the indicated antibodies. (**B**) HEK-293T cells were transfected with an increasing amount of Flag-VP2 (0, 0.25 ,0.5 or 1 ug) expressing plasmids for 24 h, the cells were lysed and analyzed by western blotting using the specified antibodies. (**C**) HEK-293T cells were transfected with Flag-tagged Vec or Flag-VP2 expressing plasmids, the LC3 puncta were observed by confocal laser scanning microscopy, with VP2 stained in red (anti-Flag) and LC3 in green. Nuclei were stained with DAPI (blue). Fluorescence intensity profiles of the red and green fluorescent signals along two different colored cross-section lines of an enlarged merged IFA image are shown. (**D**) HEK-293T cells were transfected with Flag-tagged cargo receptors (NBR1, OPTN, CALCOCO2~~NDP52~~, ~~p62~~SQSTM1, and ~~Tollip~~TOLLIP) in the presence or absence of Flag-VP2. At 24 hpi the cells were

analyzed by western blotting using the specified antibodies. (**E**) The Flag-tagged cargo receptors (NBR1, OPTN, CALCOCO2~~NDP52~~, ~~p62~~SQSTM1, and ~~Tollip~~TOLLIP) were transfected into HEK-293T cells for 24 h, followed by SVA infection for another 12 h. Immunoprecipitation was performed using anti-VP2 antibody. (**F**) Colocalization of CALCOCO2~~NDP52~~ (red) and VP2 (green) in SVA-infected HEK-293T cells was evaluated by IFA. Nuclei were stained with DAPI (blue). (**G**) *CALCOCO2~~NDP52~~*$^{-/-}$ HEK-293T cells were transfected with empty vector or Flag-VP2 expressing plasmids for 24 h, and the expression of ~~IKKε~~IKBKE, VP2, CALCOCO2~~NDP52~~ was then detected by western blotting. (**H-J**) WT or *CALCOCO2~~NDP52~~*$^{-/-}$ HEK-293T cells were infected with SVA (MOI=0.5) for 0, 3, 6, 9, or 12 h. Cell lysates were analyzed by western blotting (**H**), the mRNA expression of SVA 3D was analyzed by qPCR (**I**), and viral titers were determined from cell supernatants (**J**). (**K**) WT or *CALCOCO2~~NDP52~~*$^{-/-}$ HEK-293T cells were transfected with p~~P~~oly(I:C) for 12 h, then infected with SVA for another 12 h, the cell total RNA was extracted to detect the mRNA levels of *IFNB~~-β~~*, *~~ISG54~~IFIT2*, *~~ISG56~~IFIT1*, and *OAS1* by qPCR. All experiments were repeated for three times, yielding consistent outcomes. *P<0.05, **P< 0.01, ***P<0.001, ****P<0.0001, ns, not significant.~~.~~

Figure 4. SVA VP2 enhances K33-linked ubiquitination of ~~IKKε~~IKBKE at the K490 residue. (**A**) HEK-293T cells were co-transfected with HA-~~IKKε~~IKBKE and increasing amounts of Flag-VP2 expressing plasmids, followed by treatment with 3-MA. The immunoprecipitated proteins and WCL were subjected to western blotting analysis using the indicated antibodies. (**B**) HA-~~IKKε~~IKBKE and empty vector or

Flag-VP2 expressing plasmids were co-transfected into HEK-293T cells for 30 h, and the cells were then cultured for another 6 h in the presence of 3-MA. The cell lysates were immunoprecipitated by anti-HA antibody and subjected to western blotting analysis. (**C**) HEK-293T cells were co-transfected with HA-IKKεIKBKE or IKKεIKBKE lysine mutants along with Flag-VP2 or Flag-empty vector for 24 h. The expression of HA-IKKεIKBKE and IKKεIKBKE lysine mutants was detected by western blotting. (**D**) Ubiquitination of IKKεIKBKE and IKKεIKBKE mutants (IKKεIKBKE-^{K61R}, IKKεIKBKE-^{K490R}) was analyzed by co-transfecting HA-IKKεIKBKE, HA-IKKεIKBKE-^{K61R}, or HA-IKKεIKBKE-^{K490R} with empty vector or Flag-VP2 in the presence of 3-MA. The immunoprecipitated proteins and WCL were subjected to western blotting analysis using the indicated antibodies.

Figure 5. RNF114 is the E3 ligase that mediates IKKεIKBKE ubiquitination induced by SVA VP2. (**A**) HEK-293T cells were transfected with HA-IKKεIKBKE and MycMYC-Flag-tagged E3 ubiquitin ligases (TRIM17, TRIM25, TRIM28, RNF114 or TRIP12) for 36 h. The cell lysates were immunoprecipitated with anti-HA antibodies, and then analyzed by western blotting using the indicated antibodies. (**B**) Empty vector, Flag-tagged RNF114 or TRIM28 were transfected into HEK-293T cells. RNF114 and TRIM28 were stained with anti-Flag (green), and IKKεIKBKE was stained red. Colocalization was visualized by IFA. Nuclei were stained with DAPI (blue). (**C**) HEK-293T cells were transfected with MycMYC-Flag-tagged empty vector, TRIM28, or RNF114, in the presence or absence of Flag-VP2. At 24 hpi, cell lysates were analyzed by western blotting using the indicated antibodies. (**D**)

HEK-293T cells were transfected with NC siRNA or RNF114 siRNA for 36 h, followed by co-transfection with HA-~~IKKε~~IKBKE and Flag-Vec or Flag-VP2. The expression of the indicated proteins was detected by western blotting. (**E**) Increasing amounts of Flag-VP2 were transfected into HEK-293T cells. The expression of endogenous RNF114 was detected by western blotting. (**F**) HEK-293T cells were co-transfected with HA-~~IKKε~~IKBKE, ~~Myc~~MYC-Flag-RNF114, and increasing amounts of Flag-VP2 expressing plasmids in the presence of 3-MA. Cell lysates were subjected to co-immunoprecipitation with anti-HA antibodies and western blotting analyses using the indicated antibodies. (**G-I**) HEK-293T cells were transfected with siRNA NC or siRNA RNF114 for 36 h, followed by mock infection or SVA infection for 4, 8, and 12 h. Expression of ~~IKKε~~IKBKE, SVA 3D and VP2 was detected by western blotting (**G**), the mRNA levels of SVA 3D were analyzed by qPCR (**H**), and viral titers were determined from cell supernatants (**I**). (J) HEK-293T cells were transfected with NC siRNA or RNF114 siRNA for 24 h, then transfected with p~~P~~oly(I:C) 12 h and infected with SVA for another 12 h. The mRNA levels of *IFN-~~β~~B*, *~~ISG54~~IFIT2*, *~~ISG56~~IFIT1*, and *OAS1* were quantified by qPCR. All experiments were repeated for three times, yielding consistent outcomes. **P*<0.05, ***P*< 0.01, ****P*<0.001, *****P*<0.0001.

Figure 6. Regions (aa 1-80 and aa 201-284) of VP2 interacts with ~~IKKε~~IKBKE and mediates its degradation. (**A**) Schematic representation of Flag-tagged truncated VP2 mutants. (**B**) HEK-293T cells were transfected with 1 µg of indicated Flag-VP2 mutants for 24 h. Expression of ~~IKKε~~IKBKE was detected by western blotting. (**C**)

~~Co~~co-IP analysis to assess the interaction between HA-~~IKKε~~IKBKE and Flag-VP2 or the indicated VP2 mutants. (**D, E**) HEK-293T cells were co-transfected with *IFN~~-β~~B* (50 ng) or *ISRE* (50 ng) luciferase reporter plasmids, along with an internal control plasmid PRL-TK (5 ng) and Vec or the indicated Flag-VP2 mutants expressing plasmids (50 ng) for 24 h. SeV was used to activate the *IFN~~-β-~~B*(**D**) or *ISRE* promoter (**E**) for another 16 h. Luciferase activity was measured using the dual-luciferase assay system. (**F**) Schematic representation of HA-tagged truncated ~~IKKε~~IKBKE mutants. (**G**) Detection the expression of HA-~~IKKε~~IKBKE and HA-~~IKKε~~IKBKE mutants in HEK-293T cells with Flag-VP2 overexpression. (**H**) HEK-293T cells were transfected with Flag-VP2 and HA-~~IKKε~~IKBKE or HA-~~IKKε~~IKBKE mutants for 36 h. Cell lysates were subjected to western blotting analysis with indicated antibody.

Figure 7. Prediction and verification of key interaction residues between ~~IKKε~~ IKBKE and SVA-VP2. (**A**) The interaction model between ~~IKKε~~IKBKE and SVA-VP2 was constructed using the ZDOCK online service. Predicted interacting amino acids between ~~IKKε~~IKBKE and VP2 were highlighted using PyMOL software. (**B**) The interaction between VP2 and ~~IKKε~~IKBKE mutants was assessed in HEK-293T cells by co-transfecting HA-tagged ~~IKKε~~IKBKE or its mutants (E148A, S303A, L306A, E355A, Q368A, T374A, S377A, L707A, N708A, R709A, V710A) with Flag-tagged VP2 expressing plasmids. Cell lysates were immunoprecipitated with anti-Flag antibodies and subjected to western blotting analysis. (**C**) HEK-293T cells were transfected with HA-~~IKKε~~IKBKE or HA-~~IKKε~~IKBKE mutant (E148A) and Flag-~~v~~Vec or Flag-VP2, at 24 hpt the expression of ~~IKKε~~IKBKE was detected by

western blotting. (**D**) The interaction between ~~IKKε~~IKBKE and VP2 mutants was assessed in HEK-293T cells by co-transfecting Flag-tagged VP2 or its mutants (T16A, Q27A, C34A, D39A-T41A, R210A, G251A-T253A, E257A, R263A) with HA-tagged ~~IKKε~~IKBKE expressing plasmids. Cell lysates were immunoprecipitated with anti-HA antibodies and subjected to western blotting analysis. (**E**) Expression of ~~IKKε~~IKBKE was detected with the overexpression of Flag-VP2 or Flag-VP2 mutants through western blotting.

Figure 8. Picornavirus VP2 proteins broadly degrade ~~IKKε~~IKBKE and inhibit type I IFN signaling pathway. (**A**) HEK-293T cells were transfected with 50 ng of *IFNB* ~~*-β*~~ (left plane) or *ISRE* (right plane) reporter plasmids, 5 ng of PRL-TK plasmids, and 50 ng of SVA-VP2, FMDV-VP2, or EV71-VP2 expressing plasmids for 24 h, followed by SeV stimulate for 16 h. Luciferase activity was measured using the dual-luciferase assay system. (**B**) HEK-293T cells were transfected Vec, SVA-VP2, FMDV-VP2 or EV71-VP2 expressing plasmids for 24 h. Cell lysates were subjected to western blotting analysis using the indicated antibodies. (**C**) HEK-293T cells were co-transfected with HA-~~IKKε~~IKBKE and Vec, SVA-VP2, FMDV-VP2 or EV71-VP2 expressing plasmids, at 36 hpt, the cell lysates were immunoprecipitated with anti-HA antibodies and analyzed by western blotting. (**D**) PK-15 cells were transfected Vec, SVA-VP2 or FMDV-VP2 for 24 h. Cell lysates were subjected to western blotting analysis using indicated antibodies. (**E**) Empty vector, SVA-VP2 or FMDV-VP2 were transfected into PK-15 cells for 36 h, and the cell lysates were subjected to western blotting analysis**.** (**F**) WT or *ATG7*$^{-/-}$ HEK-293T cells were transfected Vec, SVA-VP2

or EV71-VP2 expressing plasmids for 24 h. Cell lysates were analyzed by western blotting using the indicated antibodies. (**G**) Empty vector, SVA-VP2 or FMDV-VP2 expressing plasmids were transfected into WT or *ATG5*$^{-/-}$ PK-15 cells for 24 h. The cell lysates were then analyzed by western blotting using the indicated antibodies. (**H**) WT or *CALCOCO2*~~*NDP52*~~$^{-/-}$ HEK-293T cells were transfected with Flag-Vec or Flag-EV71-VP2 expressing plasmids. At 24 hpt, the cell lysates were analyzed by western blotting using the indicated antibodies. (**I**) PK-15 cells were transfected with NC siRNA or *CALCOCO2*~~*NDP52*~~ siRNA for 36 h, then transfected with Flag-Vec or Flag-FMDV-VP2 expressing plasmids for another 24 h. The expression of ~~IKKε~~IKBKE, CALCOCO2~~NDP52~~, and Flag-VP2 was analyzed by western blotting using the specified antibodies.

Figure 9. Autophagy is crucial in picornavirus replication. (**A, B**) WT or *ATG7*$^{-/-}$ HEK-293T cells were mock-infected or infected with SVA (MOI=0.5) for 12 h. The expression of SVA-3D, ~~IKKε~~IKBKE and ATG7 was detected by western blotting **(A)**, and viral titers were determined from cell supernatants **(B)**. (**C, D**) WT or *ATG7*$^{-/-}$ HEK-293T cells were infected with EV71 (MOI=2) for 12 h, the cell lysates were analyzed by western blotting (**C**), and viral titers were determined from cell supernatants (**D**). (**E, F**) WT or *ATG5*$^{-/-}$ PK-15 cells were mock-infected or infected with SVA (MOI=2) for 18 h, the expression of SVA-3D, ~~IKKε~~IKBKE and ATG5 was detected by western blotting (**E**), and viral titers were determined from cell supernatants (**F**). (**G, H**) WT or *ATG5*$^{-/-}$ PK-15 cells were mock-infected or infected with FMDV (MOI=0.1) for 12 h. Cell lysates were analyzed by western blotting using

indicated antibodies (**G**), and viral titers were determined from cell supernatants (**H**). (**I, J**) WT or *CALCOCO2NDP52*$^{-/-}$ HEK-293T cells were mock-infected or infected with EV71 for 12 h. The expression of IKKεIKBKE, CALCOCO2NDP52 and EV71-3D was analyzed by western blotting using the indicated antibodies (**I**), and viral titers were determined from cell supernatants (**J**). (**K, L**) PK-15 cells were transfected with NC siRNA or *CALCOCO2NDP52* siRNA for 36 h, then infected with FMDV for another 12 h. The cell lysates were analyzed by western blotting (**K**), and viral titers were determined from cell supernatants (**L**).

Figure 10. The mechanisms by picornavirus VP2 protein targets IKKεIKBKE, impeding the activation of type I IFN signaling cascade, and facilitating viral replication. Upon SVA infection of host cells, the viral VP2 protein recruits RNF114, promoting the K33-linked polyubiquitination of IKKεIKBKE at specific lysine residues (K490), thereby forming the VP2-RNF114-IKKεIKBKE complex. The complex preferentially interacts with the selective autophagic receptor CALCOCO2NDP52, which recognizes the K33-linked ubiquitinated IKKεIKBKE and delivers it to autophagosomes for degradation. Upon the infection with FMDV or EV71, the VP2 protein of these viruses employs a similar strategy to recruit CALCOCO2NDP52 and deliver IKKεIKBKE to autophagosomes for degradation, thereby promoting viral replication.
