# Supplementary material for: Picornavirus VP2 protein suppresses innate immunity through selective autophagic degradation of IKBKE/IKKε
Source: Autophagy. 2025 Dec 14;22(2):330–50. doi: 10.1080/15548627.2025.2597460 (PMC12834145; doi:10.1080/15548627.2025.2597460)
Supplement: Supplementary figures.docx [file KAUP_A_2597460_SM2514.docx]

**Supplementary figures**

**
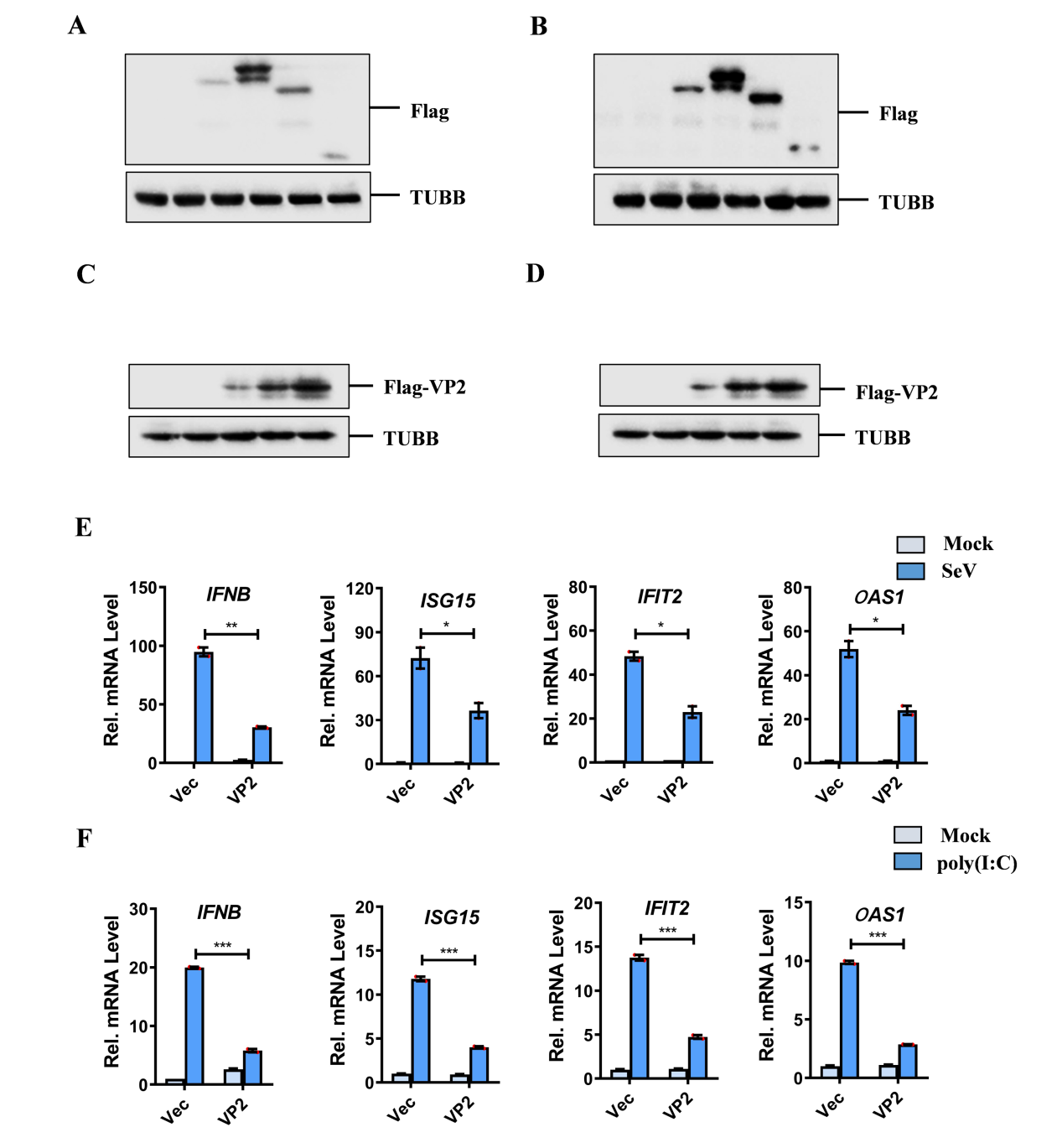
**

**Figure S1.** SVA VP2 protein blocks type I IFN signaling pathway. (**A, B**) The expression of SVA structural proteins related to the main Figure 1A (**A**) and Figure 1B (**B**) was verified by western blotting. (**C, D**) The expression of SVA VP2 protein related to the main Figure 1C (**C**) and Figure 1D (**D**) was verified by western blotting. (**E, F**) PK-15 cells were transfected with empty vector or Flag-VP2 expressing plasmids, followed by treatment with SeV (**E**) or poly(I:C) (**F**). The mRNA level of *IFNB,* *ISG15*, *IFIT2/ISG54*, and *OAS1* were detected by qPCR. All experiments were repeated for three times, yielding consistent outcomes. **P*<0.05, ***P*<0.01, ****P*<0.001.

**
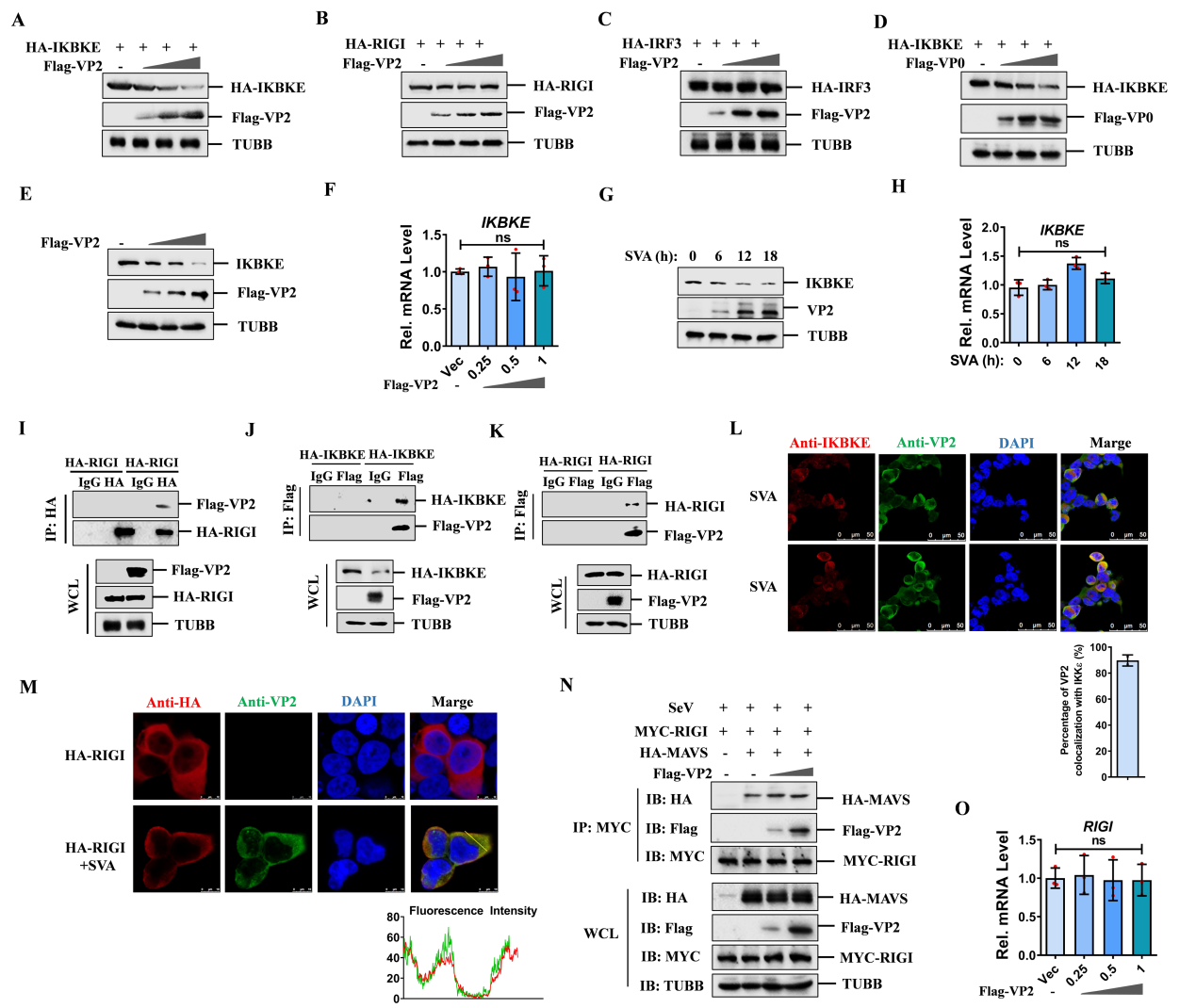
**

**Figure S2.** SVA VP2 protein inhibits the expression and interacts with IKBKE. (**A-C**) HEK-293T cells were transfected with 0, 0.25, 0.5 or 1 μg of Flag-VP2 expressing plasmids and 1μg of HA-IKBKE (**A**), HA-RIGI (**B**), or HA-IRF3 (**C**) expressing plasmids for 24 h. The cell lysates were analyzed by western blotting using the indicated antibodies. (**D**) HEK-293T cells were transfected with Flag-VP0 (0, 0.25, 0.5 or 1 μg) expressing plasmids and 1μg of HA-IKBKE for 24 h. The expression of IKBKE was analyzed by western blotting. (**E, F**) PK-15 cells were transfected with Flag-VP2 expressing plasmids (0, 0.25, 0.5 or 1 μg) for 24 h. The expression of IKBKE was analyzed by western blotting (**E**), and the mRNA levels of *IKBKE* were detected by qPCR (**F**). **(G, H)** PK-15 cells were mock-infected or infected with SVA (MOI=1) for 6, 12 or 18 h. The protein expression levels of IKBKE were detected by western blotting **(G)**, and the mRNA levels of *IKBKE* were analyzed by qPCR **(H).** **(I)** HEK-293T cells were co-transfected with HA-RIGI and Flag-vec or Flag-VP2 expressing plasmids. The cell lysates were immunoprecipitated with anti-HA antibodies and subjected to western blotting analysis. (**J, K**) HEK-293T cells were co-transfected with empty vector or Flag-VP2 and HA-IKBKE (**J**) or HA-RIGI (**K**) expressing plasmids for 36 h. The cell lysates were immunoprecipitated with anti-Flag or control IgG antibodies and subjected to western blotting analysis. (**L**) The low magnification and statistical analysis of VP2 and IKBKE colocalization. (**M**) HEK-293T cells were transfected with HA-RIGI for 12 h, followed by mock-infection or infection with SVA at an MOI of 0.1 for an additional 10 h. The subcellular localization of HA-RIGI and SVA-VP2 was evaluated by IFA. The nuclei were stained by DAPI (blue). The fluorescence for VP2 (green) and HA-RIGI (red) was detected. (**N**) HEK-293T cells were co-transfected with MYC-RIGI, HA-MAVS and increasing amounts of Flag-VP2 expressing plasmids for 24 h, then treated with SeV for another 12 h. The cell lysates were immunoprecipitated with anti-MYC antibodies and subjected to western blotting analysis. (**O**) HEK-293T cells were transfected with an increasing amounts Flag-VP2 expressing plasmids (0, 0.25, 0.5 or 1 μg) for 24 h. The mRNA levels of *RIGI* were analyzed by qPCR.

**
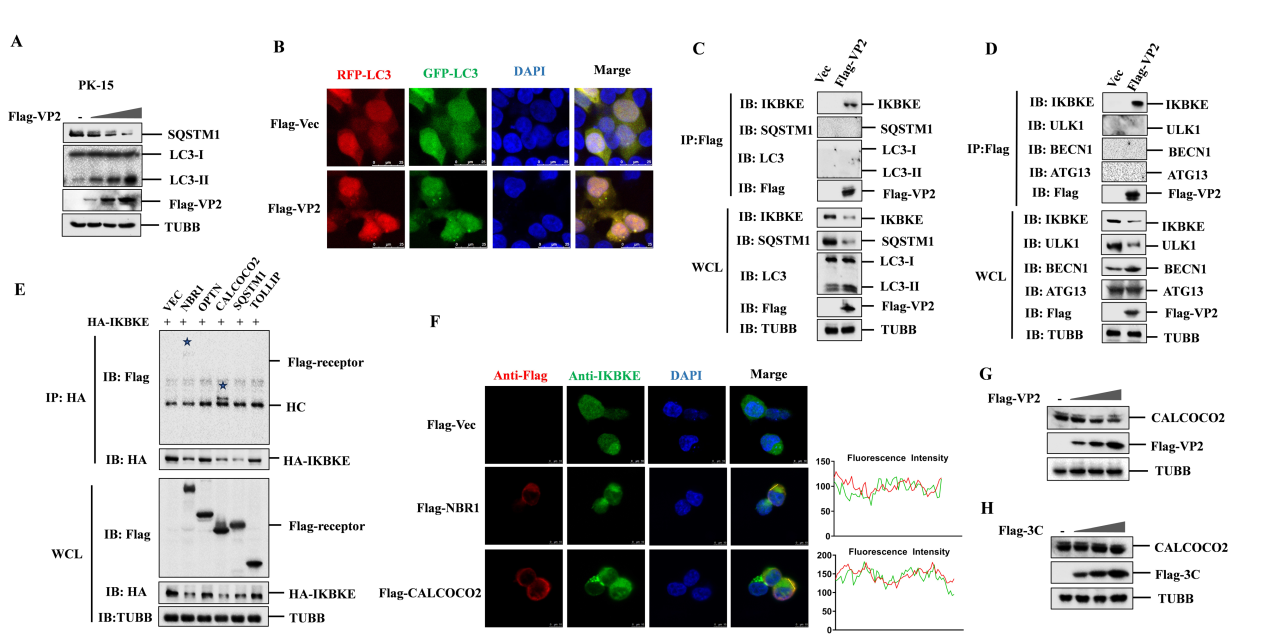
**

**Figure S3.** SVA VP2 promotes the degradation of IKBKE through the CALCOCO2-mediated selective autophagy. (**A**) PK-15 cells were transfected with an increasing amounts Flag-VP2 expressing plasmids (0, 0.25, 0.5 or 1 μg) for 24 h. The cell lysates were analyzed by western blotting using the indicated antibodies. (**B**) HEK-293T cells were co-transfected with RFP-GFP-LC3 and empty vector or Flag-VP2 expressing plasmids. The localization of RFP-LC3 (red) and GFP-LC3 (green) were evaluated by IFA with nuclei stained by DAPI (blue). (**C**) HEK-293T cells were transfected with empty vector or Flag-VP2. The cell lysates were immunoprecipitated with anti-Flag antibodies and subjected to western blotting analysis. The interaction between SVA VP2 and SQSTM1 or LC3 was then analyzed. (**D**) HEK-293T cells were transfected with empty vector or Flag-VP2 expressing plasmids. The cell lysates were immunoprecipitated with anti-Flag antibodies and subjected to western blotting analysis. The interaction between SVA VP2 and canonical autophagic proteins (ULK1, ATG13 or BECN1) was then analyzed. (**E**) HEK-293T cells were transfected with empty vector or autophagy receptor proteins (NBR1, OPTN, CALCOCO2, SQSTM1, or TOLLIP) and HA-IKBKE for 36 h. The cell lysates were immunoprecipitated with anti-HA antibody and subjected to western blotting using the indicated antibodies. (**F**) HEK-293T cells were transfected with empty vector, Flag-NBR1, or Flag-CALCOCO2 for 24 h. The subcellular localization of IKBKE and Flag-NBR1 or Flag-CALCOCO2 was evaluated by IFA, with Flag-NBR1 and Flag-CALCOCO2 stained in red (anti-Flag) and IKBKE in green. The nuclei were stained by DAPI (blue). (**G, H**) HEK-293T cells were transfected with Flag-VP2 (**G**) or Flag-3C (**H**) expressing plasmids (0, 0.25, 0.5 or 1 μg) for 24 h. The expression of CALCOCO2 was detected by western blotting.


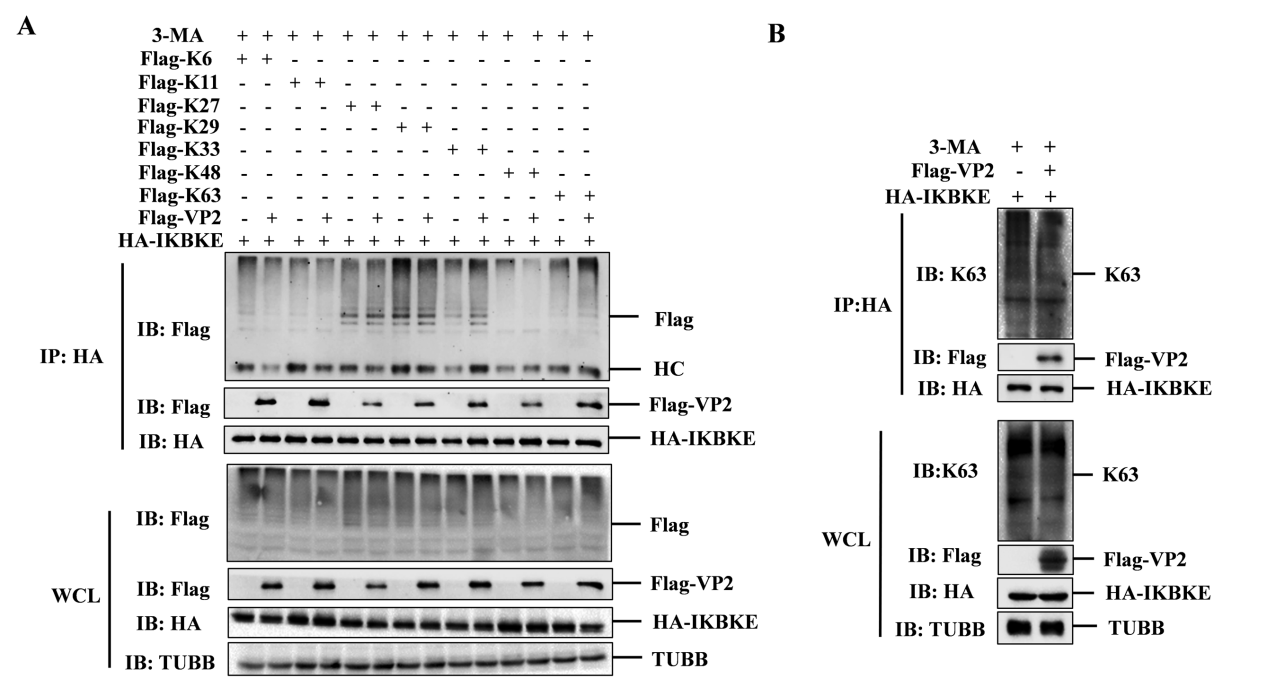


**Figure S4.** SVA VP2 enhances the K33-linked ubiquitination of IKBKE. (**A**) HEK-293T cells were co-transfected with HA-IKBKE, various Flag-ubiquitin mutants (K6-, K11-, K27-, K29-, K33-, K48-, or K63- only), and Flag vector or Flag-VP2 plasmids for 30 h, followed by a 6 h treatment with 3-MA. The cell lysates were immunoprecipitated with anti-HA antibody. The immunoprecipitated proteins and WCL were subjected to western blotting analysis using the indicated antibodies, and the ubiquitination of IKBKE was then analyzed. (**B**) HEK-293T cells were co-transfected with HA-IKBKE and Flag vector or Flag-VP2 for 30 h, followed by a 6 h treatment with 3-MA. The cell lysates were immunoprecipitated with anti-HA antibody. The immunoprecipitated proteins and WCL were subjected to western blotting analysis using the indicated antibodies.


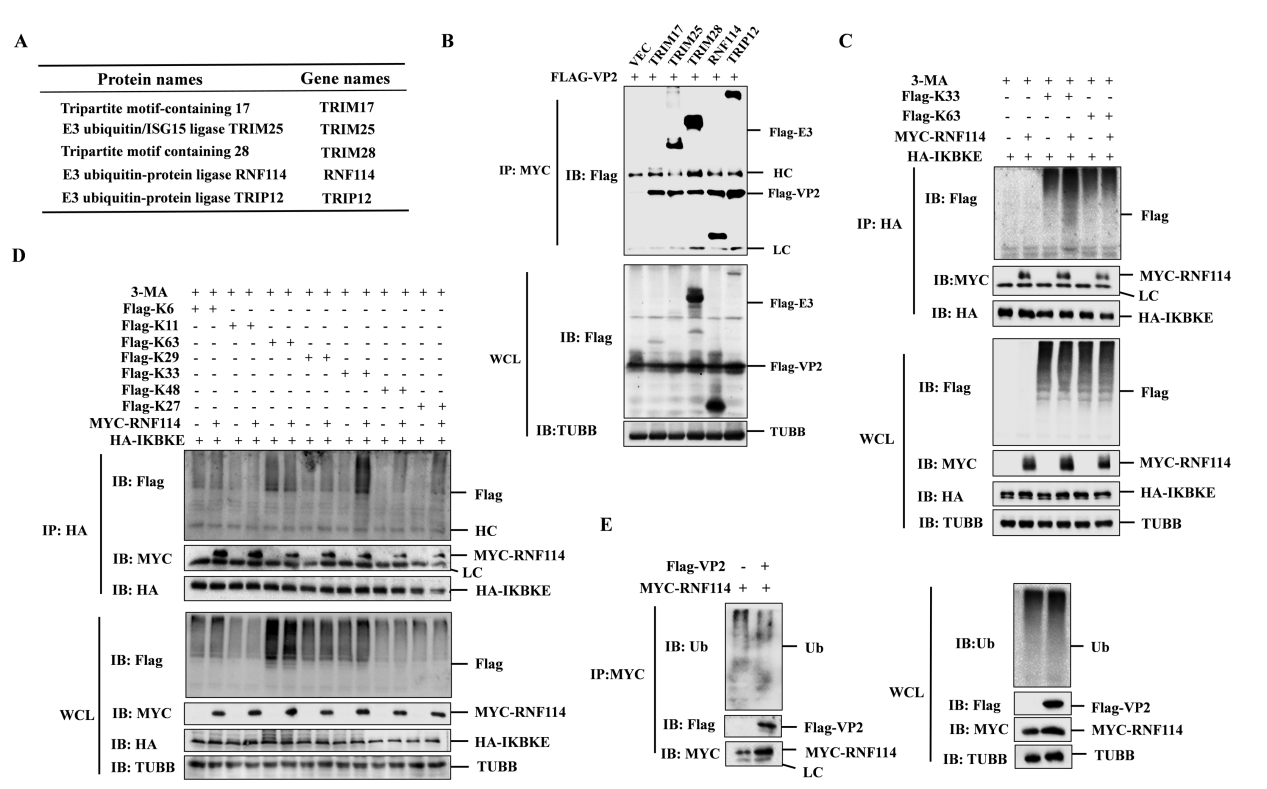


**Figure S5.** RNF114 catalyzes polyubiquitination of IKBKE induced by SVA VP2. (**A**) HEK-293T cells were transfected with Flag vector or Flag-VP2 expressing plasmids for 24 h. The cell lysates were then immunoprecipitated with anti-Flag antibody, and the immunoprecipitated proteins were determined by mass spectrometry method. The identified cellular proteins (E3 ubiquitin proteins) that potentially interact with VP2 were listed. (**B**) HEK-293T cells were co-transfected with empty vector or Flag-VP2 and MYC-Flag-tagged E3 ubiquitin ligases (TRIM17, TRIM25, TRIM28, RNF114 or TRIP12) for 36 h. The cell lysates were immunoprecipitated with anti-MYC antibodies and subjected to western blotting analysis using the indicated antibodies. (**C**) HEK-293T cells were co-transfected with HA-IKBKE, MYC-Flag-RNF114 and Flag-K33 or Flag-K63 expressing plasmids for 30 h, followed by 3-MA treatment for another 6 h. The cell lysates were immunoprecipitated with anti-HA antibodies and subjected to western blotting analysis using the indicated antibodies. (**D**) HA-IKBKE, various Flag-ubiquitin plasmids (K6-, K11-, K27-, K29-, K33-, K48-, or K63- only), and MYC-Flag vector or MYC-Flag-RNF114 plasmids were transfected into HEK-293T cells for 30 h, followed by 3-MA and MG132 treat another 6 h. The cell lysates were immunoprecipitated with anti-HA antibody. (**E**) HEK-293T cells were co-transfected with MYC-Flag-RNF114 and Flag vector or Flag-VP2 for 36 h, then the cell lysates were immunoprecipitated with anti-MYC antibody to detect the ubiquitination of RNF114.


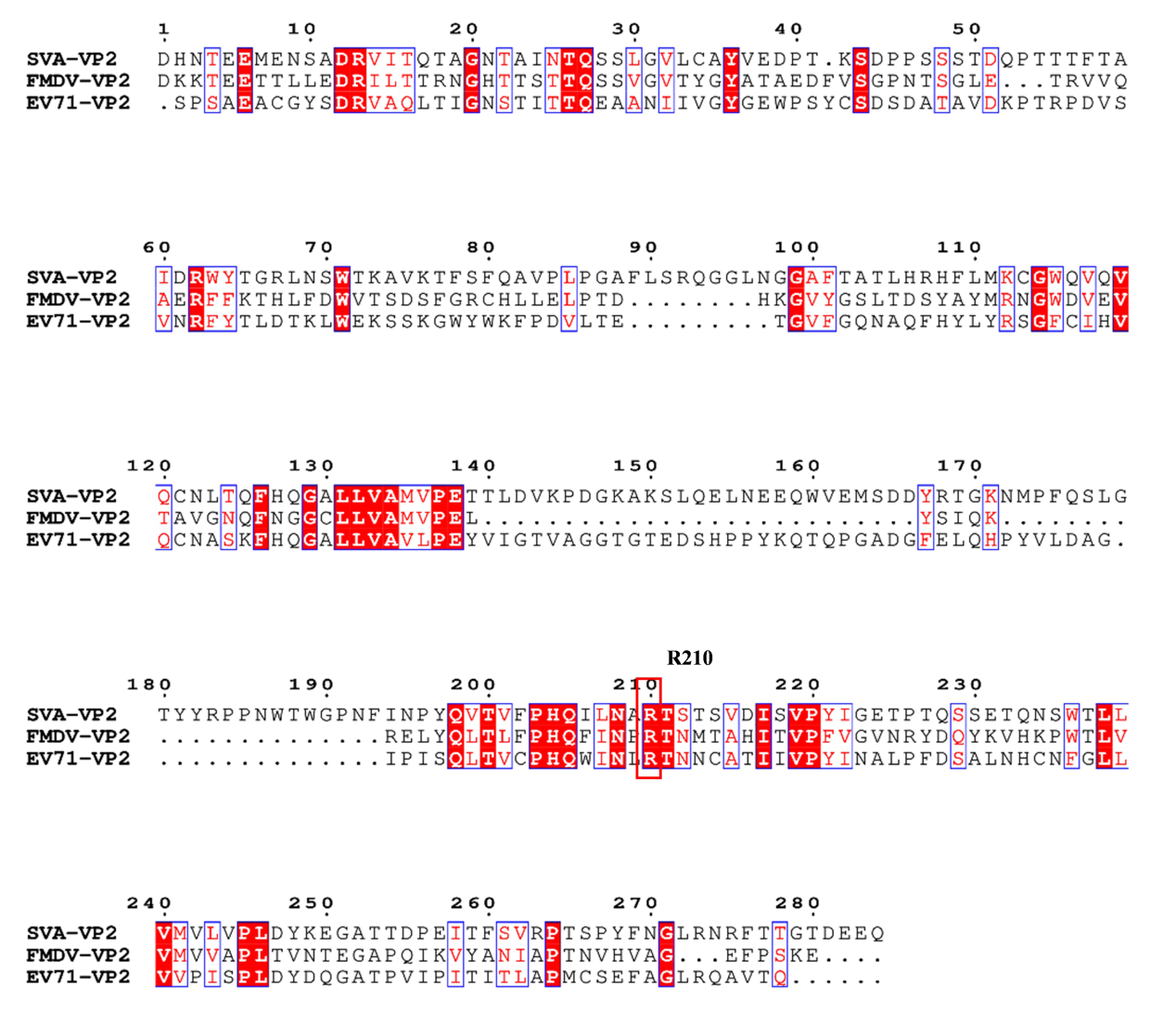


**Figure S6.** Alignment of SVA, FMDV, and EV71 VP2 protein sequences. The residues R210 were marked by red blocks. The strictly conserved residues are indicated by red blocks, and similar residues are enclosed in blue boxes.


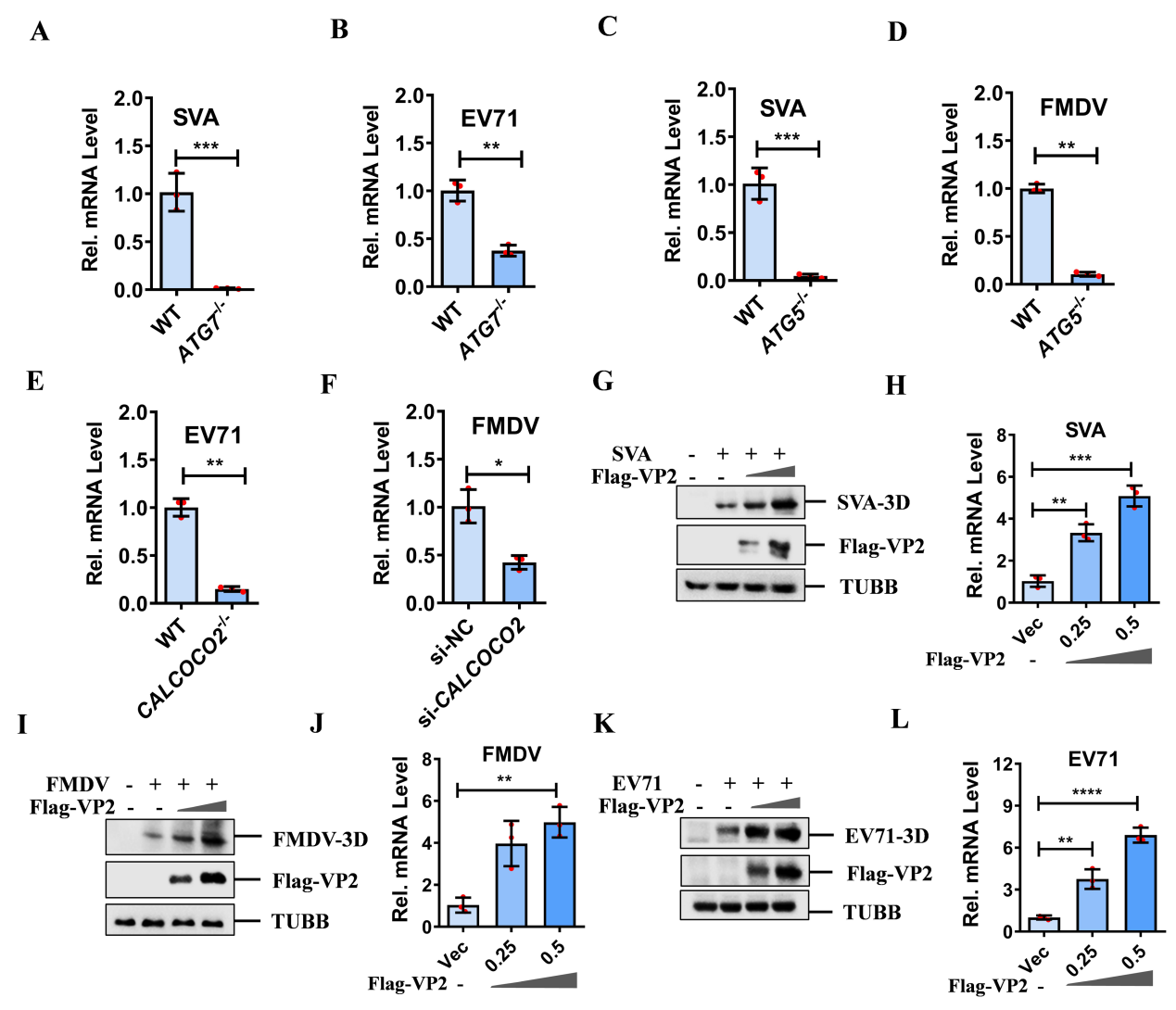


**Figure S7.** Autophagy is crucial in picornavirus replication. (**A, B**) WT or *ATG7*^-/-^ HEK-293T cells were mock-infected or infected with SVA (**A**) or EV71 (**B**) for 12 h. Total RNAs were extracted, and the viral mRNA levels were determined by qPCR. (**C, D**) WT or *ATG5*^-/-^ PK-15 cells were mock-infected or infected with SVA (MOI=2) for 18 h (**C**) or FMDV (MOI=0.1) for 12 h (**D**). Total RNAs were extracted, and the viral mRNA levels were determined by qPCR. (**E**) WT or *CALCOCO2*^-/-^ HEK-293T cells were mock-infected or infected with EV71 for 12 h. Total RNAs were extracted, and the viral 3D mRNA levels were determined by qPCR. (**F**) PK-15 cells were transfected NC siRNA or *CALCOCO2* siRNA for 36 h, followed by FMDV infection for another 12 h. Total RNAs were extracted, and the viral 3D mRNA levels were determined by qPCR. (**G, H**) HEK-293T cells were transfected with SVA Flag-VP2 (0, 0, 0.5 or 1 ug) for 24 h, followed by infection with SVA for an additional 12 h. The viral replication was analyzed by western blotting with SVA 3D antibodies **(G)**, the cellular total RNA was extracted and analyzed by qPCR using SVA 3D gene **(H)**. (**I, J**) PK-15 cells were transfected with increasing amounts of FMDV Flag-VP2 expressing plasmids for 24 h, followed by FMDV infection for an additional 10 h. The cells were lysed and analyzed by western blotting with the indicated antibodies **(I)**, the cellular total RNA was extracted and analyzed by qPCR **(J)**. (**K, L**) EV71 Flag-VP2 (0, 0, 0.5 or 1 ug) expressing plasmids were transfected into HEK-293T cells for 24 h, followed by infection with EV71 for another 12 h. The viral replication was analyzed by western blotting **(K)** and qPCR **(L)**.
